# Supplementary material for: Golgi apparatus-targeted aggregation-induced emission luminogens for effective cancer photodynamic therapy
Source: Nat Commun. 2022 Apr 21;13:2179. doi: 10.1038/s41467-022-29872-7 (PMC9023483; doi:10.1038/s41467-022-29872-7)
Supplement: Supplementary file 1 — Supplementary Information [file 41467_2022_29872_MOESM1_ESM.pdf]

Golgi apparatus-targeted aggregation-induced emission luminogens for effective cancer photodynamic therapy

Minglun Liu[1], Yuncong Chen[1,2]*, Yan Guo[1], Hao Yuan[1], Tongxiao Cui[1], Shankun Yao[1], Suxing Jin[1], Huanhuan Fan[1], Chengjun Wang[3], Ran Xie[1], Weijiang He[1,2]*, Zijian Guo[1,2]*

1. State Key Laboratory of Coordination Chemistry, School of Chemistry and Chemical Engineering, Chemistry and Biomedicine Innovation Center (ChemBIC), Nanjing University, Nanjing 210023, China. Email: chenyc@nju.edu.cn; heweij69@nju.edu.cn; zguo@nju.edu.cn

2. Nanchuang (Jiangsu) Institute of Chemistry and Health, Nanjing 210000, China

3. Sinopec Shengli Petroleum Engineering Limited Company, Dongying 257068, China.

Supplementary Methods

Synthesis and characterization parts

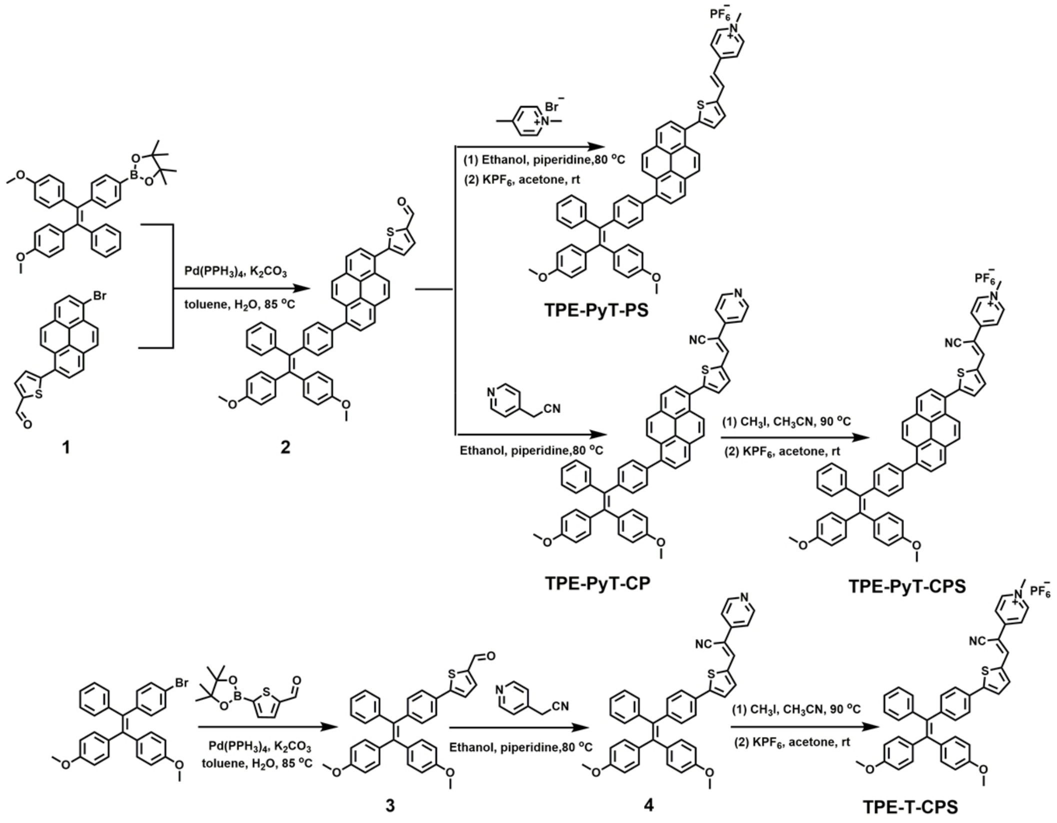

Supplementary Figure 1. Syntheses routes of **TPE-PyT-CPS**, **TPE-PyT-CP**, **TPE-T-CPS** and **TPE-PyT-PS**.

Syntheses of compound 1.

Under N_2 atmosphere, to a mixture of 1,6-dibromopyrene (5.0 g, 13.90 mmol), 5-(4,4,5,5-tetramethyl-1,3,2-dioxaborolan-2-yl)thiophene-2-carbaldehyde (3.33 g, 14.0 mmol) and potassium carbonate (4.14 g) in toluene (90 mL) and water (15 mL) was added Tetrakis(triphenylphosphine)palladium (0.81 g, 0.70 mmol) in one portion, then the mixture was heated to 85 ^{o}C for over 18 hours. After completion, the mixture was cooled to room temperature and removal of the solvent. The resulting residue was extracted with dichloromethane and the crude product was purified by column chromatography using DCM and petroleum ether (1/1, R_f = 0.4) as the eluent to give a brown solid (2.3 g, 42.3 %). ^{1}H NMR (400 MHz, Chloroform-d) δ 10.02 (s, 1H), 8.49 (d, *J* = 9.2 Hz, 1H), 8.42 (d, *J* = 9.2 Hz, 1H), 8.27 (d, *J* = 8.2 Hz, 1H), 8.24 (d, *J* = 7.9 Hz, 1H), 8.17 (d, *J* = 9.3 Hz, 1H), 8.11 (d, *J* = 7.9 Hz, 1H), 8.06 (d, *J* = 9.3 Hz, 1H), 8.03 (d, *J* = 8.2 Hz, 1H), 7.92 (d, *J* = 3.8 Hz, 1H), 7.47 (d, *J* = 3.8 Hz, 1H). ^{13}C NMR (101 MHz, Chloroform-d) δ 182.90, 152.61, 144.21, 136.68, 131.68, 130.65, 130.20, 129.88, 129.30, 128.97, 128.88, 128.80, 128.68, 128.35, 127.06, 125.99, 125.77, 125.20, 124.49, 124.35, 120.87, 29.70. HR-MS (ESI, positive mode, m/z): calcd for $C_{21}H_{11}BrOS$ [M+H]$^{+}$:390.9787, found: 390.9779.

Syntheses of compound 2.

Under N_2 atmosphere, to a mixture of compound **1** (0.942 g, 2.0 mmol), 2-(4-(1,2-bis(4-methoxyphenyl)-2-phenylvinyl)phenyl)-4,4,5,5-tetramethyl-1,3,2-dioxaborolane (1.10g, 2.1 mmol), potassium carbonate (0.55 g) in toluene (15 mL) and water (3 mL) was added Tetrakis(triphenylphosphine)palladium (0.115 g, 0.095 mmol) in one portion, then the mixture was heated to 85 ^{o}C for over 18 hours. After completion, the mixture was cooled to room temperature and removal of the solvent. The resulting residue was extracted with dichloromethane and the crude product was purified by column chromatography using petroleum ether and DCM (1/1, R_f = 0.3) as the eluent to give a brown solid (0.38 g, 27.1 %). ^{1}H NMR (400 MHz, Chloroform-d) δ 10.02 (s, 1H), 8.44 (d, *J* = 9.2 Hz, 1H), 8.24 (d, *J* = 7.9 Hz, 1H), 8.21 (d, *J* = 4.5 Hz, 1H), 8.19 (d, *J* = 3.2 Hz, 1H), 8.14 (d, *J* = 9.3 Hz, 1H), 8.10 (d, *J* = 7.9 Hz, 1H), 8.02 (t, *J* = 8.7 Hz, 2H), 7.93 (d, *J* = 3.9 Hz, 1H), 7.51 (d, *J* = 3.8 Hz, 1H), 7.38 (d, *J* = 8.1 Hz, 2H), 7.24 – 7.13 (m, 7H), 7.07 (d, *J* = 8.7 Hz, 2H), 7.01 (d, *J* = 8.7 Hz, 2H), 6.74 (d, *J* =8.7 Hz, 2H), 6.68 (d, *J* = 8.7 Hz, 2H), 3.81 (s, 3H), 3.77 (s, 3H). ^{13}C NMR (101 MHz, Chloroform-d) δ 182.92, 171.99, 158.15, 156.79, 153.26, 144.18, 143.96, 143.56, 140.65, 136.75, 136.44, 132.77, 132.64, 131.80, 131.50, 131.41, 130.06, 129.95, 129.14, 128.74, 128.21, 128.13, 127.82, 127.14, 126.58, 126.25, 125.19, 124.45, 124.05, 113.08, 113.05, 55.20, 55.12. HR-MS (ESI, positive mode, m/z): calcd for $C_{49}H_{34}O_3S$ $[M+H]^+$:703.2301, found: 703.2296.

Syntheses of TPE-PyT-CP.

Compound **2** (147 mg, 0.228 mmol) and 2-(pyridin-4-yl)acetonitrile (34 mg, 0.228 mmol) were added to 50 ml flasks under nitrogen protection, then 15 ml ethanol and 4 drops of piperidine were added. The mixture was heated to 80 ^{o}C and stirred for 24 hours. After the reaction, dichloromethane was used to extract and then anhydrous magnesium sulfate was used to dry. Further separation using column to separate the crude products with DCM (R_f = 0.4) yielded 120 mg red solid with a yield of 65.3%. ^{1}H NMR (400 MHz, Chloroform-d) δ 8.72 (d, *J* = 5.5 Hz, 2H), 8.48 (d, *J* = 9.2 Hz, 1H), 8.27 – 8.18 (m, 3H), 8.18 – 8.12 (m, 2H), 8.07 – 7.99 (m, 3H), 7.94 (d, *J* = 3.8 Hz, 1H), 7.76 (d, *J* = 5.0 Hz, 2H), 7.55 (d, *J* = 3.8 Hz, 1H), 7.38 (d, *J* = 8.1 Hz, 2H), 7.25 – 7.13 (m, 7H), 7.07 (d, *J* = 8.8 Hz, 2H), 7.01 (d, *J* = 8.6 Hz, 2H), 6.75 (d, *J* = 8.8 Hz, 2H), 6.68 (d, *J* = 8.7 Hz, 2H), 3.81 (s, 3H), 3.77 (s, 3H). ^{13}C NMR (101 MHz, Chloroform-d) δ 158.27, 158.15, 148.72, 148.71, 144.17, 143.57, 140.66, 138.88, 138.71, 138.46, 137.85, 137.52, 136.44, 136.26, 136.05, 132.76, 132.64, 131.80, 131.80, 131.50, 131.42, 130.07, 129.95, 129.31, 129.25, 128.82, 128.73, 128.34, 128.16, 127.84, 127.82, 128.73, 127.17, 126.61, 126.25, 125.32, 125.21, 124.98, 124.51, 124.04, 119.97, 117.05, 113.09, 113.05, 55.20, 55.12. HR-MS (ESI, positive mode, m/z): calcd for $C_{56}H_{38}N_2O_2S$ $[M+H]^+$: 803.2727, found: 803.2757.

Syntheses of TPE-PyT-CPS.

A mixture of TPE-PyT-CP (105 mg, 0.13 mmol) and CH_3I (300 mg, 2.12 mmol) in CH_3CN (10 ml) was refluxed at 90°C for 4 h under nitrogen protection. After cooling to room temperature, Et_2O (15 mL) was added into the solution to form a solid. The solid was filtered off and dried under vacuum, then the obtained solid was dissolved in acetone (10 mL), and a solution of KPF_6 (915 mg, 5 mmol) in 2 mL H_2O was added. The mixture was stirred at room temperature for 6 h. Acetone was removed under reduced pressure, and the residue was purified by silica gel chromatography using DCM/MeOH (30:1, R_f = 0.5) as the eluent to give TPE-PyT-CPS as a dark

red solid (103 mg, 82.2 %). ^{1}H NMR (400 MHz, DMSO-d6) δ 9.12 (s, 1H), 9.01 (d, *J* = 6.9 Hz, 2H), 8.51 (d, *J* = 9.3 Hz, 1H), 8.42 (dd, *J* = 8.2, 2.6 Hz, 2H), 8.40 – 8.33 (m, 3H), 8.27 (dd, *J* = 14.1, 8.7 Hz, 2H), 8.20 (d, *J* = 4.0 Hz, 1H), 8.09 (dd, *J* = 17.9, 8.6 Hz, 2H), 7.87 (d, *J* = 3.9 Hz, 1H), 7.44 (d, *J* = 8.1 Hz, 2H), 7.20 (dq, *J* = 16.0, 7.4 Hz, 5H), 7.11 (d, *J* = 7.0 Hz, 2H), 7.00 (d, *J* = 8.7 Hz, 2H), 6.94 (d, *J* = 8.6 Hz, 2H), 6.81 (d, J = 8.7 Hz, 2H), 6.73 (d, J = 8.7 Hz, 2H), 4.33 (s, 3H), 3.75 (s, 3H), 3.70 (s, 3H). ^{13}C NMR (101 MHz, DMSO-d6) δ 157.94, 157.80, 151.99, 148.77, 145.61, 143.81, 143.67, 143.20, 140.51, 140.50, 140.43, 138.35, 137.90, 137.75, 137.36, 135.66, 135.54, 132.22, 132.09, 131.37, 130.98, 130.85, 130.41, 129.85, 129.69, 129.61, 129.15, 128.48, 128.42, 128.24, 128.02, 127.90, 127.56, 127.50, 126.40, 125.85, 125.66, 125.11, 124.48, 124.14, 123.85, 122.39, 116.50, 113.26, 113.21, 100.78, 55.04, 54.94, 47.19, 29.04. HR-MS (ESI, positive mode, m/z): calcd for $C_{57}H_{41}N_2O_2S^+$[M-PF_6^-] $^+$: 817.2883, found: 817.2894.

Syntheses of TPE-PyT-PS.

A mixture of TPE-PyT-CP (120 mg, 0.17 mmol) and 1,4-dimethylpyridin-1-ium bromide (300 mg, 2.12 mmol) were added to 50 ml flasks under nitrogen protection, then 20 ml ethanol and 6 drops of piperidine were added. The mixture was heated to 80 °C and stirred for 24 hours. After cooling to room temperature, Et_2O (20 ml) was added into the solution to form a solid. The solid was filtered off and dried under vacuum, then the obtained solid was dissolved in acetone (10 ml), and a solution of KPF_6 (915 mg, 5 mmol) in 2 mL H_2O was added. The mixture was stirred at room temperature for 6 h. Acetone was removed under reduced pressure, and the residue was purified by silica gel chromatography using CH_2Cl_2/MeOH (40:1, R_f = 0.5) as the eluent to give TPE-PyT-PS as a brown solid (80 mg, 50.2 %). ^{1}H NMR (400 MHz, DMSO-d6) δ 8.85 (d, *J* = 6.4 Hz, 2H), 8.55 (d, *J* = 9.2 Hz, 1H), 8.45 – 8.37 (m, 2H), 8.34 (dd, *J* = 12.7, 2.9 Hz, 2H), 8.28 – 8.15 (m, 4H), 8.07 (dd, *J* = 14.3, 8.5 Hz, 2H), 7.72 (d, *J* = 3.5 Hz, 1H), 7.63 (d, *J* = 3.5 Hz, 1H), 7.43 (d, *J* = 7.9 Hz, 2H), 7.31 (d, *J* = 16.0 Hz, 1H), 7.20 (dq, *J* = 14.9, 7.1 Hz, 5H), 7.11 (d, *J* = 7.2 Hz, 2H), 7.00 (d, *J* = 8.5 Hz, 2H), 6.94 (d, *J* = 8.4 Hz, 2H), 6.81 (d, *J* = 8.5 Hz, 2H), 6.73 (d, *J* = 8.5 Hz, 2H), 4.25 (s, 3H), 3.75 (s, 3H), 3.71 (s, 3H). ^{13}C NMR (101 MHz, DMSO-d6) δ 157.83, 157.70, 152.04, 145.11, 144.88, 143.57, 143.07, 141.34, 140.32, 138.27, 137.73, 137.55, 135.57, 135.44, 133.42, 132.61, 132.10, 131.97, 130.87, 130.74, 129.97, 129.72, 129.53, 128.61, 128.31, 128.23, 128.02, 127.91, 127.46, 126.29, 125.30, 124.92, 124.42, 124.13, 123.85, 123.08, 122.07, 113.16, 54.83, 46.74, 28.92. HR-MS (ESI, positive mode, m/z): calcd for $C_{56}H_{42}NO_2S^+$ [M] $^+$:792.2931, found: 792.2903.

Syntheses of ompound 3.

Under N_2 atmosphere, to a mixture of 4,4'-(2-(4-bromophenyl)-2-phenylethene-1,1-diyl)bis(methoxybenzene) (4.71g, 10.0 mmol), 5-(4,4,5,5-tetramethyl-1,3,2-dioxaborolan-2-yl)thiophene-2-carbaldehyde (3.33 g, 14.0 mmol) and potassium carbonate (4.14 g) in toluene (90 ml) and water (15 ml) was added Tetrakis(triphenylphosphine)palladium (0.81 g, 0.70 mmol) in one portion, then the mixture was heated to 85 °C for over 18 hours. After completion, the mixture was cooled to room temperature and removal of the solvent. The resulting residue was extracted with dichloromethane and the crude product was purified by column chromatography using DCM and petroleum ether (1/1, R_f = 0.3) as the eluent to give a brown solid (3.8 g, 75.6 %). ^{1}H NMR (400 MHz, Chloroform-d) δ 9.86 (s, 1H), 7.70 (d, J = 3.9 Hz, 1H), 7.42 (d, *J* = 8.4 Hz, 2H), 7.34 (d, *J* = 3.9 Hz, 1H), 7.16 – 7.09 (m, 3H), 7.07 (d, *J* = 8.4 Hz, 2H), 7.03 (dd, *J* = 7.6, 1.9 Hz, 2H),

6.97 (d, J = 8.8 Hz, 2H), 6.93 (d, J = 8.8 Hz, 2H), 6.67 (d, J = 8.8 Hz, 2H), 6.64 (d, J = 8.8 Hz, 2H), 3.75 (s, 3H), 3.74 (s, 3H). ^{13}C NMR (101 MHz, Chloroform-d) δ 182.71, 158.36, 158.23, 154.37, 145.95, 143.83, 141.96, 141.20, 138.16, 137.46, 135.99, 132.62, 132.59, 132.16, 131.39, 130.46, 127.86, 126.35, 125.63, 123.72, 113.22, 113.02, 55.12, 55.09. HR-MS (ESI, positive mode, m/z): calcd for $C_{33}H_{26}O_3S^+$ $[M]^+$:503.1675, found: 503.1665.

Syntheses of compound 4

Compound **3** (251 mg, 0.50 mmol) and 2-(pyridin-4-yl)acetonitrile (88 mg, 0.60 mmol) were added to 50 ml flasks under nitrogen protection, then 20 ml ethanol and 10 drops of piperidine were added. The mixture was heated to 80 ^oC and stirred for 24 hours. After the reaction, dichloromethane was used to extract and then anhydrous magnesium sulfate was used to dry. Further separation using column to separate the crude products with DCM (R_f= 0.35) as the eluent to give a red solid (210 mg, 69.7%). ^1H NMR (400 MHz, Chloroform-d) δ 8.66 (d, J = 5.3 Hz, 2H), 7.84 (s, 1H), 7.65 (d, J = 4.0 Hz, 1H), 7.58 (d, J = 6.1 Hz, 2H), 7.44 (d, J = 8.4 Hz, 2H), 7.34 (d, J = 4.0 Hz, 1H), 7.13 (d, J = 7.2 Hz, 3H), 7.06 (dd, J = 10.5, 8.1 Hz, 4H), 6.98 (d, J = 8.7 Hz, 2H), 6.94 (d, J = 8.7 Hz, 2H), 6.68 (d, J = 8.8 Hz, 2H), 6.64 (d, J = 8.8 Hz, 2H), 3.76 (s, 3H), 3.74 (s, 3H). ^{13}C NMR (101 MHz, Chloroform-d) δ 158.40, 158.25, 152.07, 149.41, 145.83, 143.84, 142.66, 141.25, 138.20, 137.43, 136.62, 136.02, 135.81, 132.62, 132.60, 132.20, 131.42, 130.36, 127.86, 126.37, 125.57, 123.78, 119.68, 117.18, 113.25, 113.03, 103.72, 55.13, 55.10. HR-MS (ESI, positive mode, m/z): calcd for $C_{40}H_{30}N_2O_2S^+$$[M+H]^+$:603.2101, found: 603.2082.

Syntheses of TPE-T-CPS

A mixture of compound **4** (200 mg, 0.33 mmol) and CH_3I (300 mg, 2.12 mmol) in CH_3CN (15 ml) was refluxed at 90 °C for 4 h under nitrogen protection. After cooling to room temperature, Et_2O (15 mL) was added into the solution to form a solid. The solid was filtered off and dried under vacuum, then the obtained solid was dissolved in acetone (10 ml), and a solution of KPF_6 (915 mg, 5 mmol) in 2 ml H_2O was added. The mixture was stirred at room temperature for 6 h. Acetone was removed under reduced pressure, and the residue was purified by silica gel chromatography using CH_2Cl_2/MeOH (30:1, R_f= 0.5) as the eluent to give TPE-T-CPS as a dark red solid (150 mg, 59.6 %). ^1H NMR (400 MHz, DMSO-d6) δ 8.97 (s, 1H), 8.96 (s, 2H), 8.30 (d, J = 6.9 Hz, 2H), 7.98 (d, J = 4.1 Hz, 1H), 7.80 (d, J = 4.0 Hz, 1H), 7.63 (d, J = 8.3 Hz, 2H), 7.15 (dq, J = 14.2, 7.0 Hz, 3H), 7.07 (d, J = 8.3 Hz, 2H), 7.00 (d, J = 6.8 Hz, 2H), 6.93 (d, J = 8.6 Hz, 2H), 6.88 (d, J = 8.6 Hz, 2H), 6.75 (d, J = 8.7 Hz, 2H), 6.70 (d, J = 8.7 Hz, 2H), 4.30 (s, 3H), 3.69 (d, J = 2.5 Hz, 6H). ^{13}C NMR (101 MHz, DMSO-d6) δ 157.92, 157.78, 153.18, 148.78, 145.69, 145.40, 143.58, 143.30, 141.27, 140.88, 137.71, 135.42, 135.28, 132.03, 131.96, 131.79, 130.74, 129.70, 129.50, 127.91, 126.40, 125.66, 125.44, 122.14, 116.38, 113.30, 113.09, 99.92, 54.84, 47.02. HR-MS (ESI, positive mode, m/z): calcd for $C_{41}H_{33}N_2O_2S^+$$[M]^+$:617.2257, found: 617.2243.

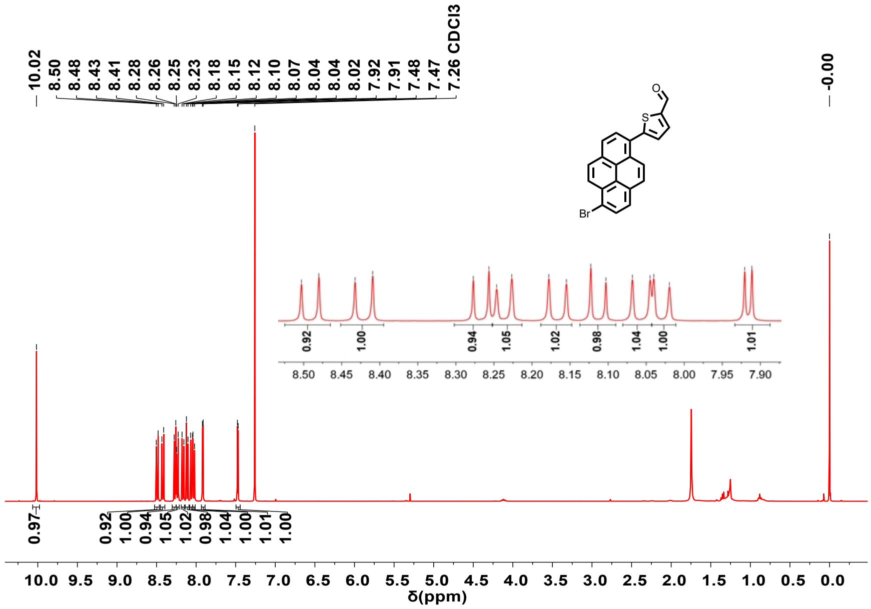

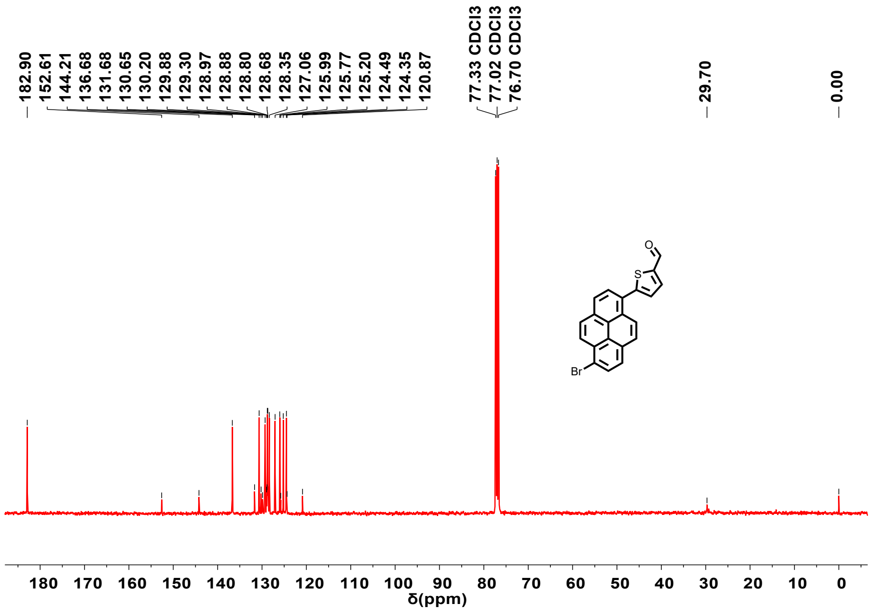

Supplementary Figure 2. ^{1}H and ^{13}C NMR spectra of compound **1** in $CDCl_3$.

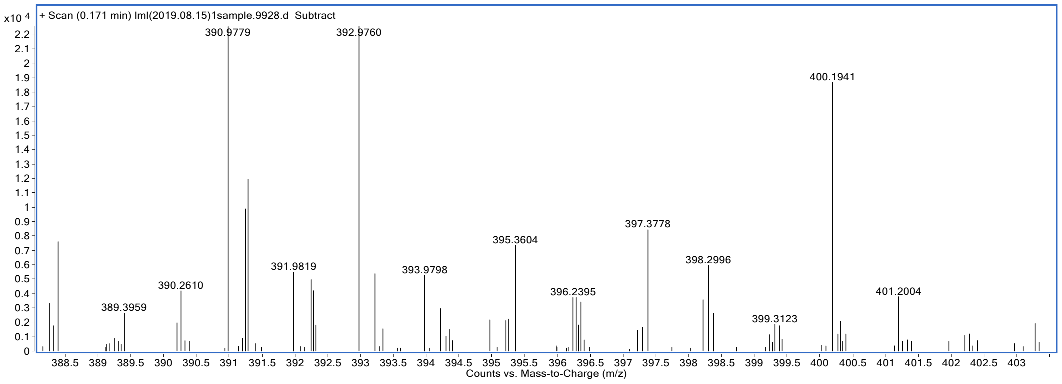

Supplementary Figure 3. HR-MS spectrum of compound **1**.

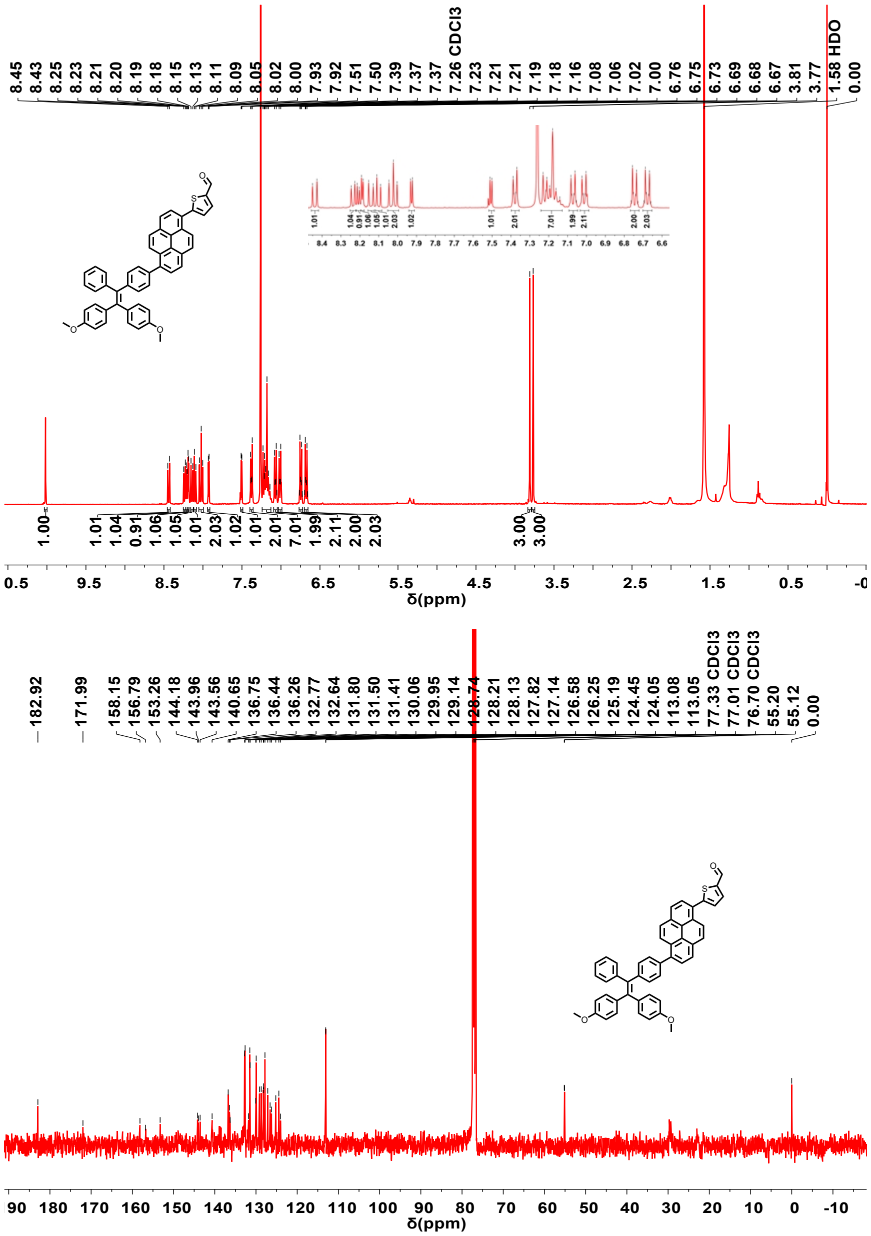

Supplementary Figure 4. ^{1}H and ^{13}C NMR spectra of compound **2** in $CDCl_3$.

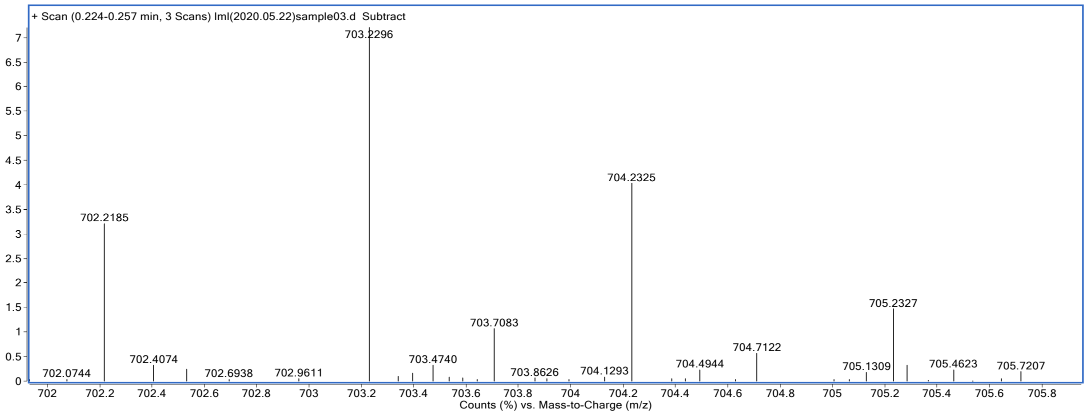

Supplementary Figure 5. HR-MS spectrum of compound **2**.

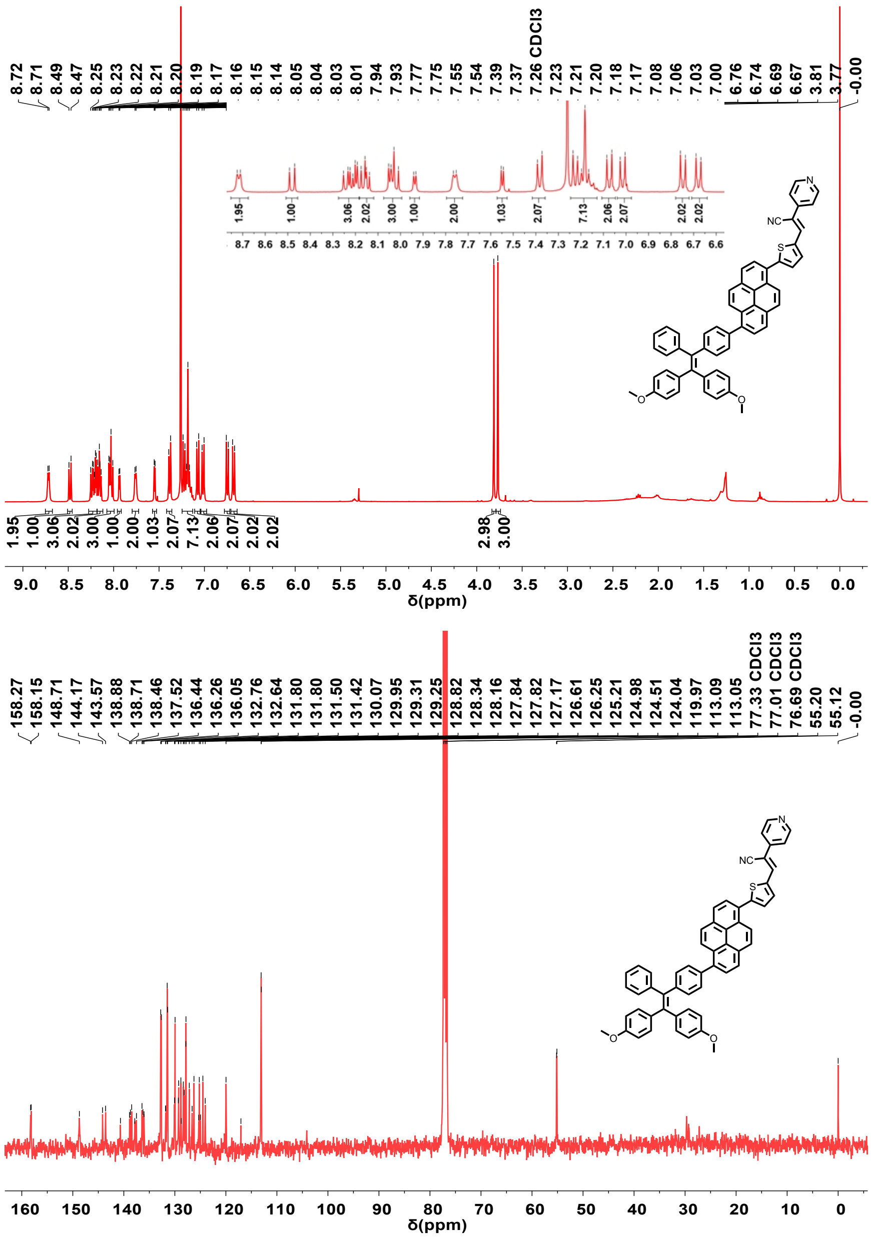

Supplementary Figure 6. ^{1}H and ^{13}C NMR spectra of **TPE-PyT-CP** in $CDCl_3$.

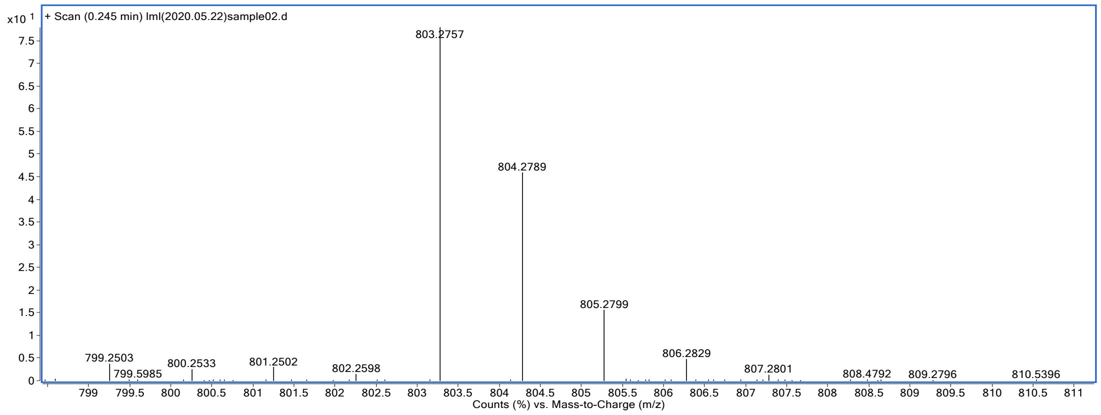

Supplementary Figure 7. HR-MS spectrum of **TPE-PyT-CP**.

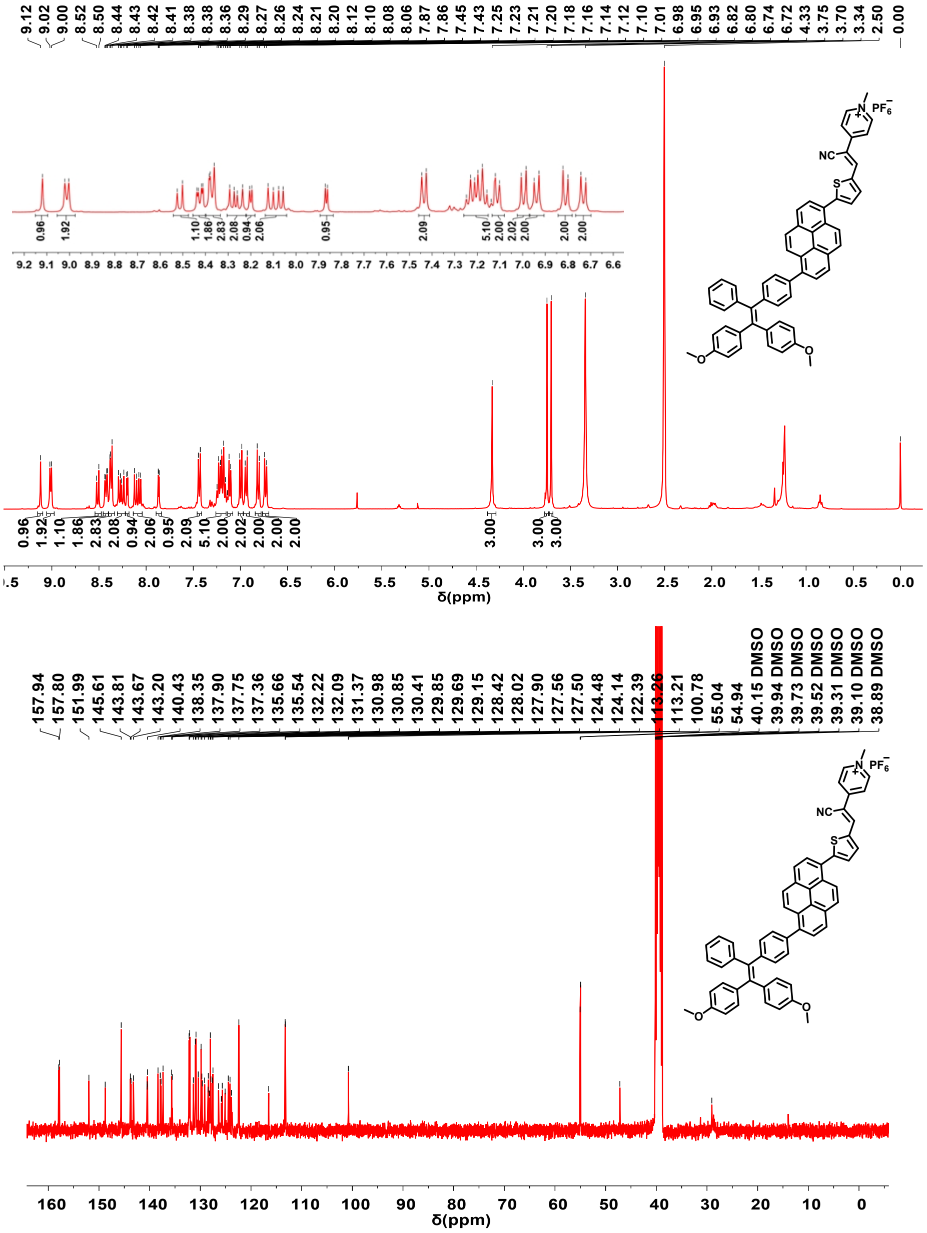

Supplementary Figure 8. ^{1}H and ^{13}C NMR spectra of **TPE-PyT-CPS** in DMSO-d6.

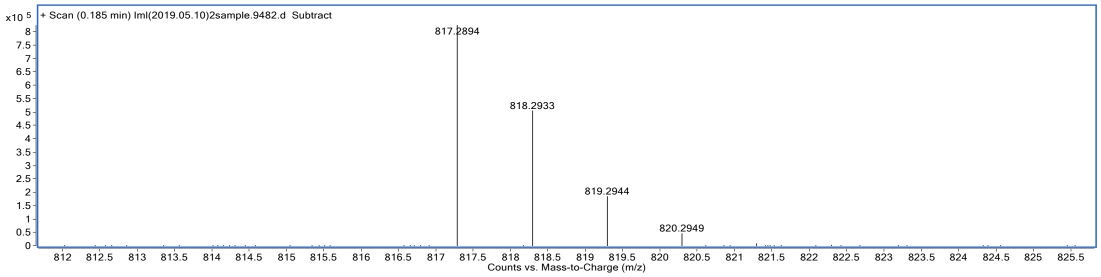

Supplementary Figure 9. HR-MS spectrum of **TPE-PyT-CPS**.

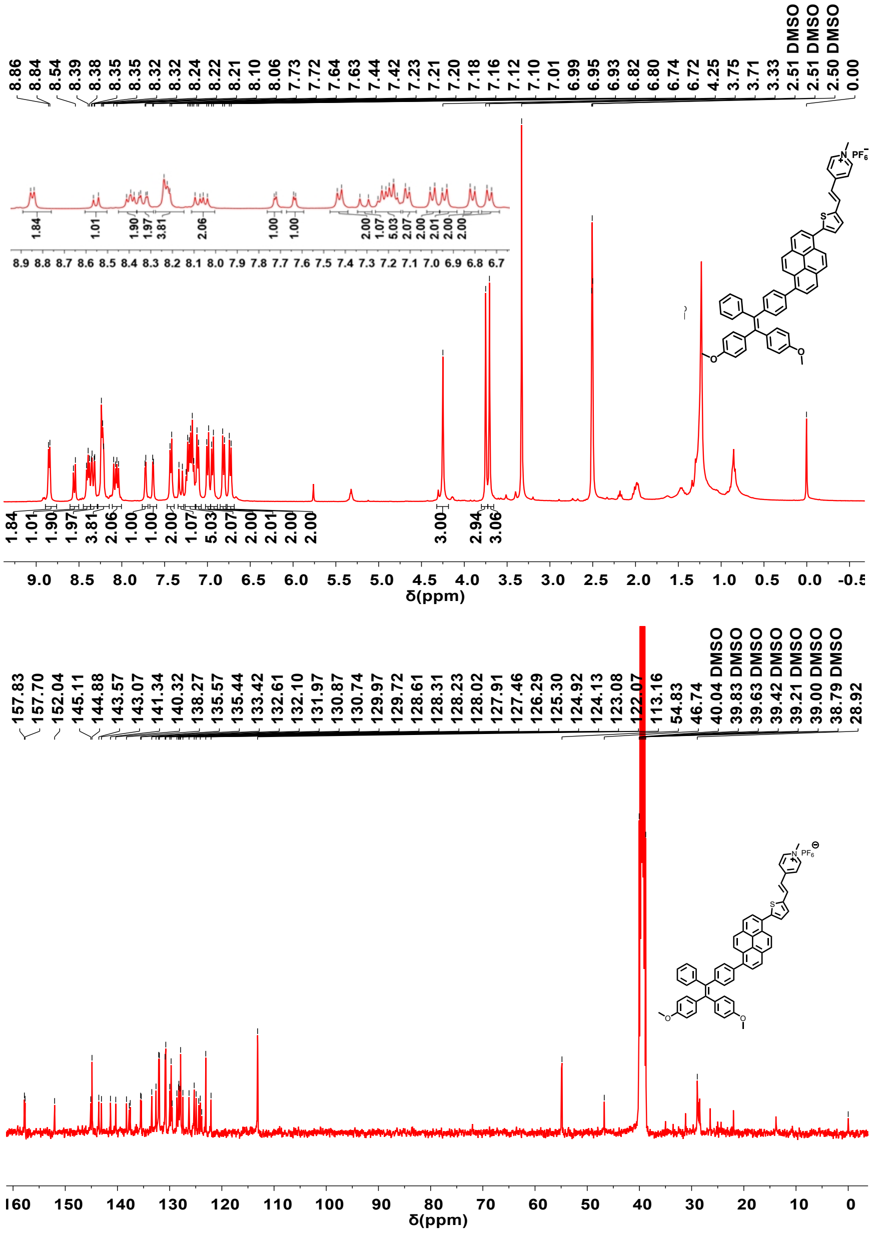

Supplementary Figure 10. ^{1}H and ^{13}C NMR spectra of **TPE-PyT-PS** in DMSO-d6.

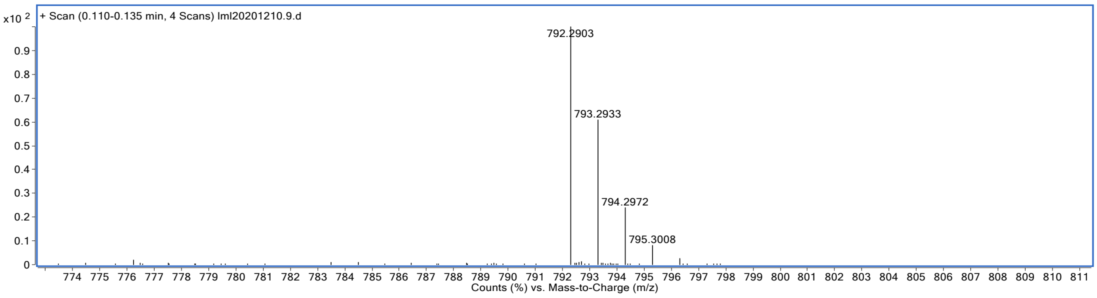

Supplementary Figure 11. HR-MS spectrum of **TPE-PyT-PS**.

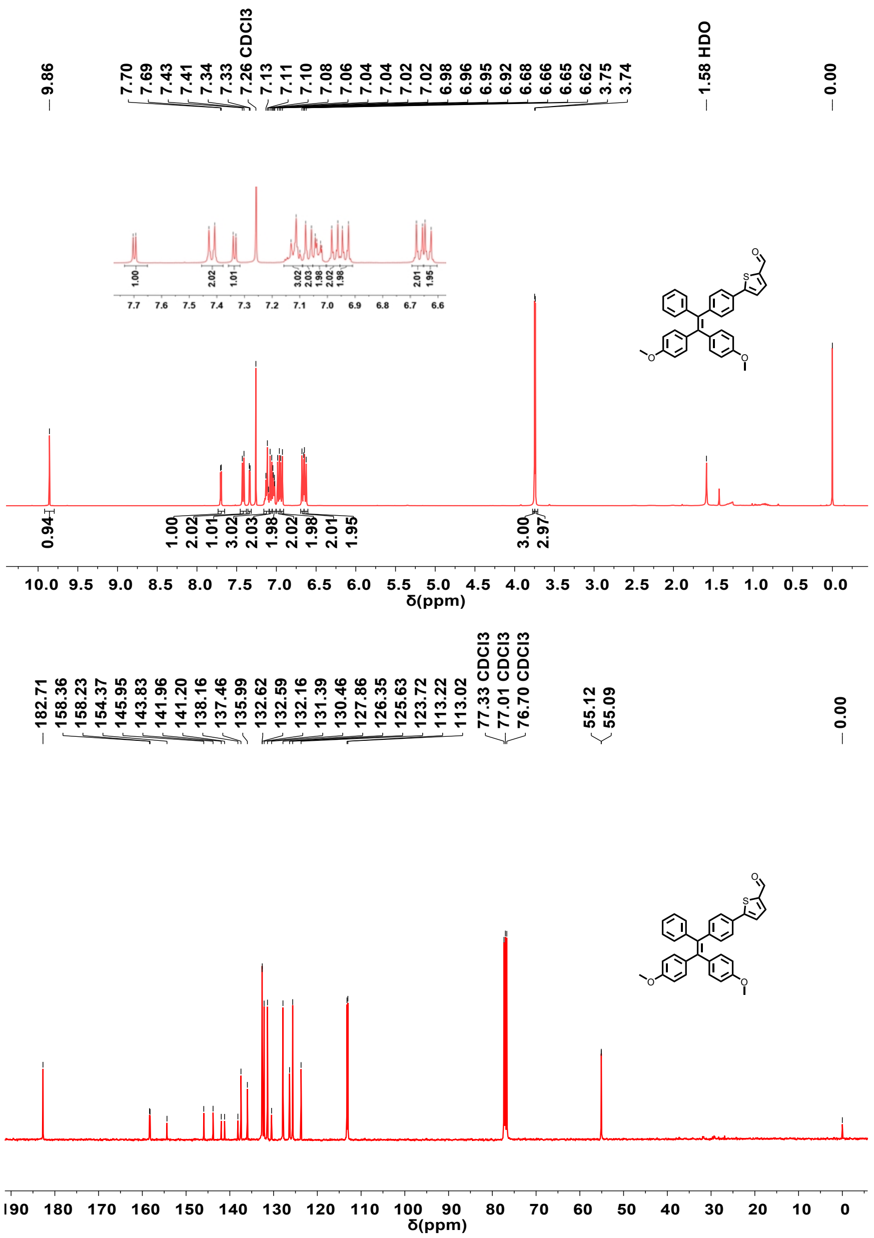

Supplementary Figure 12. ^{1}H and ^{13}C NMR spectra of compound **3** in $CDCl_3$.

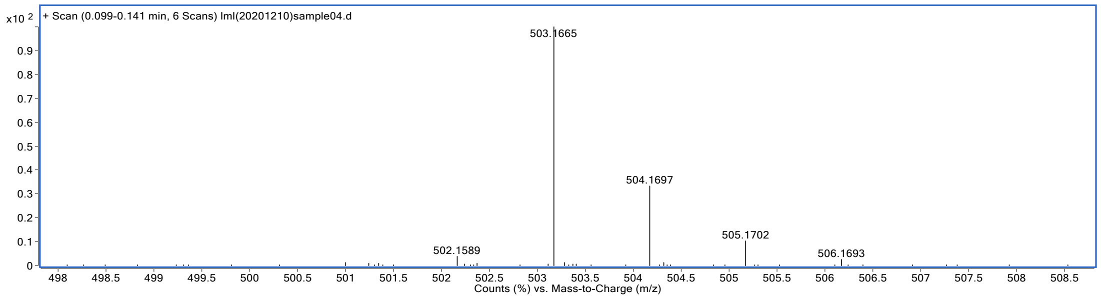

Supplementary Figure 13. HR-MS spectrum of compound **3**.

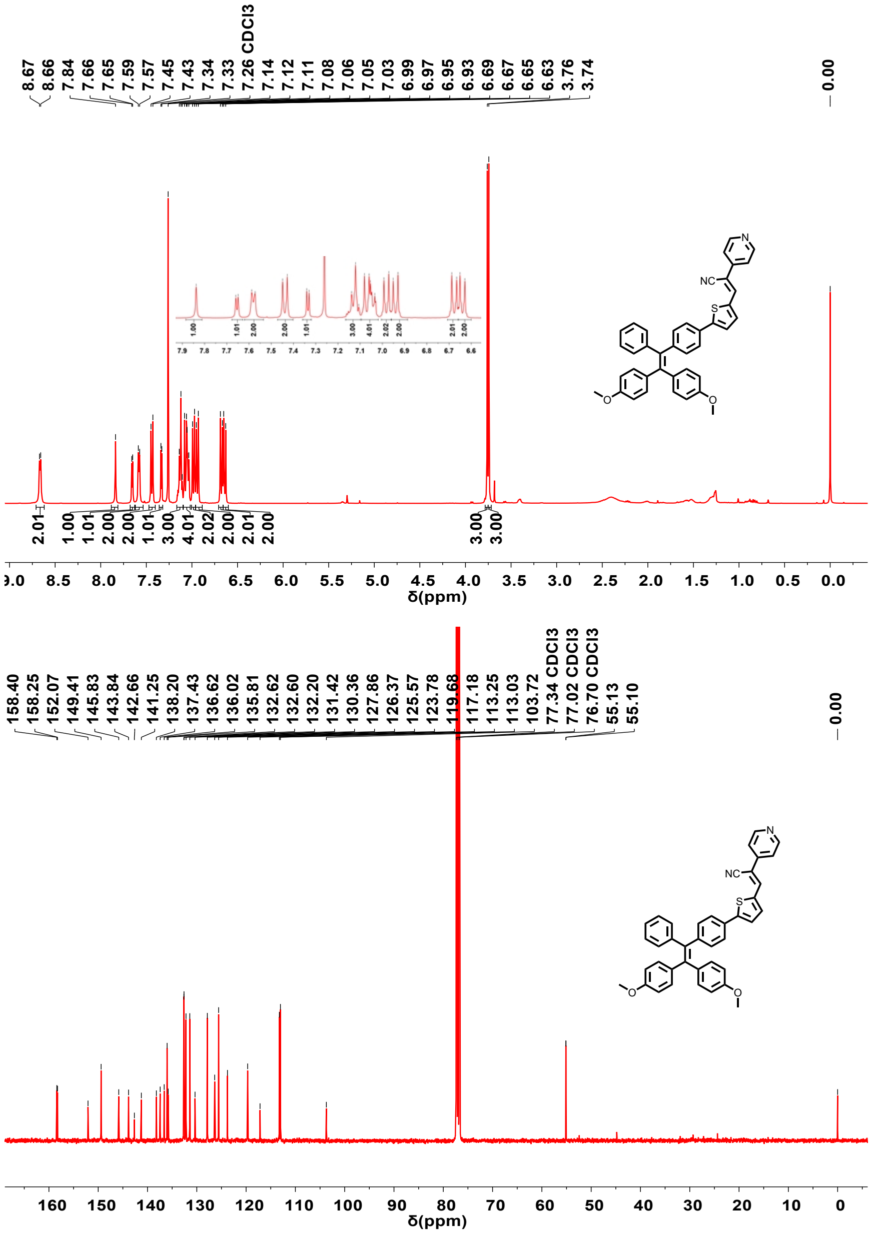

Supplementary Figure 14. ^{1}H and ^{13}C NMR spectra of **4** in $CDCl_3$.

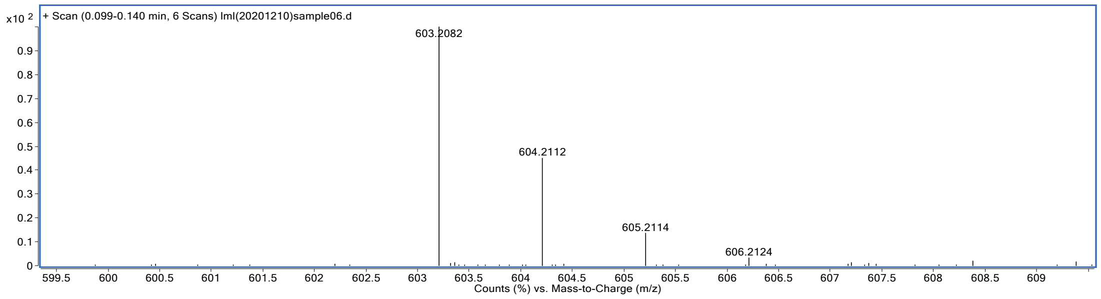

Supplementary Figure 15. HR-MS spectrum of compound **4**.

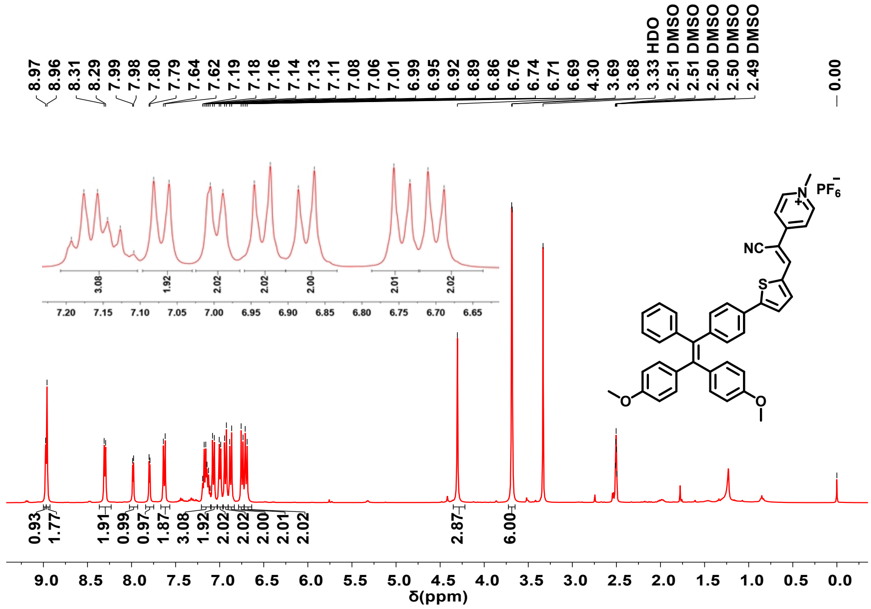

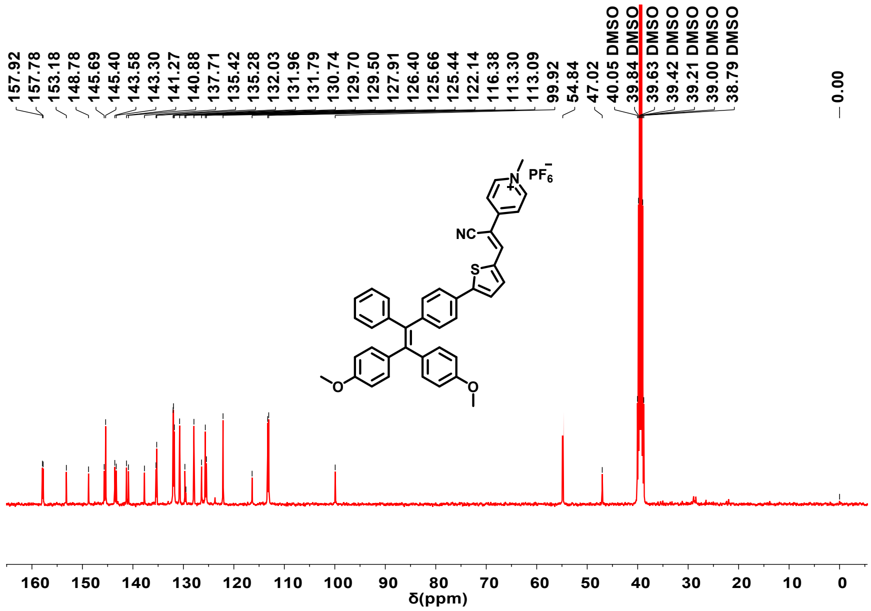

Supplementary Figure 16. ^{1}H and ^{13}C NMR spectra of **TPE-T-CPS** in DMSO-d6.

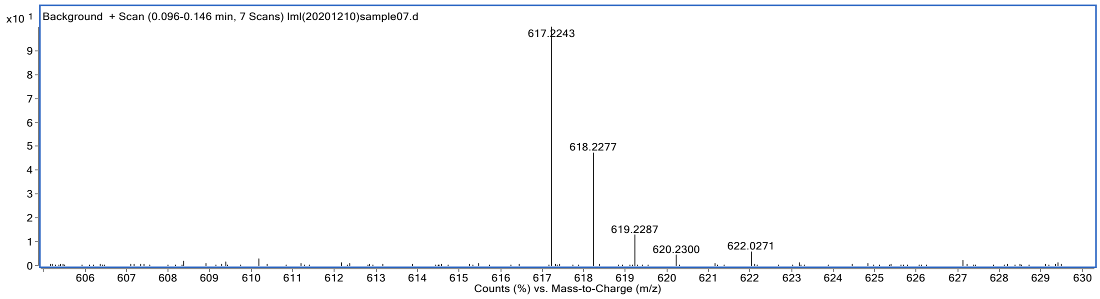

Supplementary Figure 17. HR-MS spectrum of **TPE-T-CPS**.

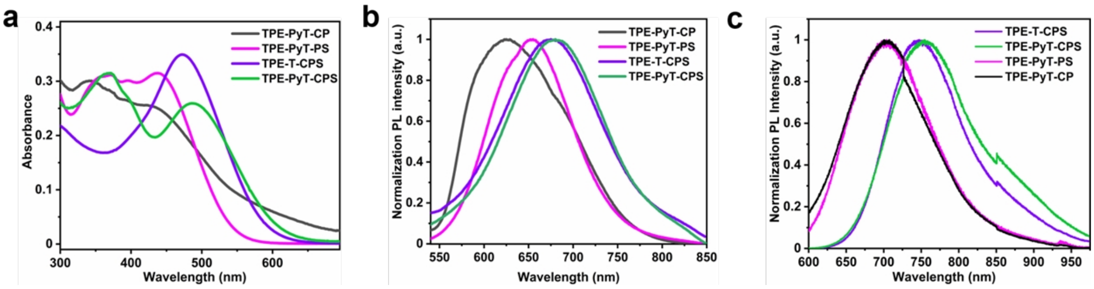

Supplementary Figure 18. **Characterization the photophysical properties of the AIEgens.** (a) Absorption and (b) emission spectra of TPE-PyT-CPS, TPE-PyT-CP, TPE-PyT-PS, TPE-T-CPS (10 μM) were recorded in water (containing 1% acetonitrile, *v/v*). (c) Emission spectra of TPE-PyT-CPS, TPE-PyT-CP, TPE-PyT-PS, TPE-T-CPS in solid state. The experiments in a-c were performed two times independently with similar results, representative images are shown. Source data are provided as a Source Data file.

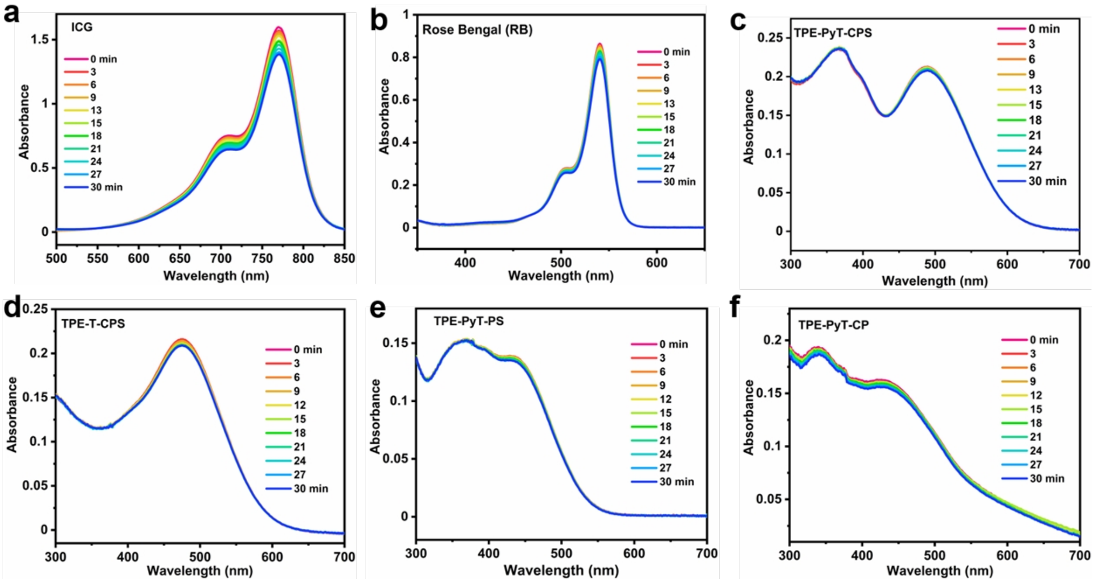

Supplementary Figure 19. **The photostability of the AIEgens compared with indocyanine green (ICG) and Rose Bengal (RB).** (a) The absorbance of the ICG (5 μM) in aqueous solution under 808 nm laser irradiation (25 mW cm^{-2}) for 30 min. (b) The absorbance of the RB (5 μM) in aqueous solution under 532 nm laser irradiation (25 mW cm^{-2}) for 30 min. (c-f) The absorbance of the AIEgens (5 μM) including TPE-PyT-CPS (c), TPE-T-CPS (d), TPE-PyT-PS (e) and TPE-PyT-CP (f) in aqueous solution under 532 nm laser irradiation (25 mW cm^{-2}) for 30 min. The experiments in a-f were performed two times independently with similar results, representative images are shown. Source data are provided as a Source Data file.

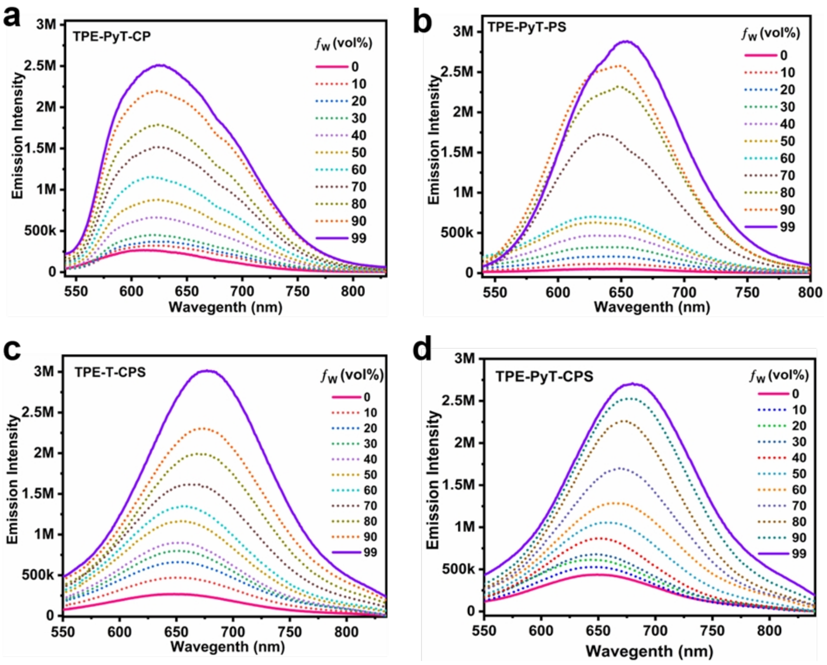

Supplementary Figure 20. **AIE characteristics of the AIEgens in aqueous solution**. Emission spectra of the AIEgens (10 μM) including TPE-PyT-CP (a), TPE-PyT-PS (b), TPE-T-CPS (c) and TPE-PyT-CPS (d) in acetonitrile/water mixtures with varied water fractions (f_w). The experiments in a-d were performed two times independently with similar results, representative images are shown. Source data are provided as a Source Data file.

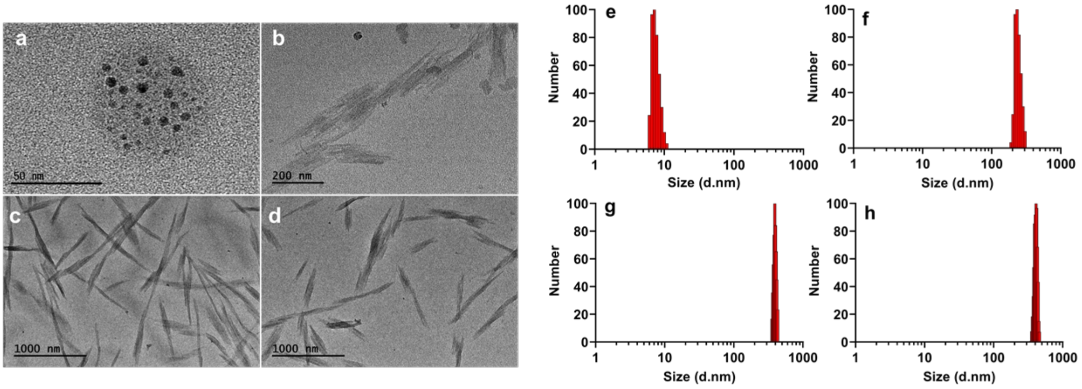

Supplementary Figure 21. **The transmission electron microscopy (TEM) and dynamic light scattering (DLS) and images of the AIEgens.** The TEM images of the AIEgens including TPE-PyT-PS (a), TPE-T-CPS (b), TPE-PyT-CP (c) and TPE-PyT-CPS (d) in aqueous solution. Scale bar: 50 nm for (a); 200 nm for (b); 1000 nm for (c) and (d). DLS images of the AIEgens including TPE-PyT-PS (e), TPE-T-CPS (f), TPE-PyT-CP (g) and TPE-PyT-CPS (h) in aqueous solution. The experiments in (a-d) were performed three times independently, representative images are shown. Source data are provided as a Source Data file.

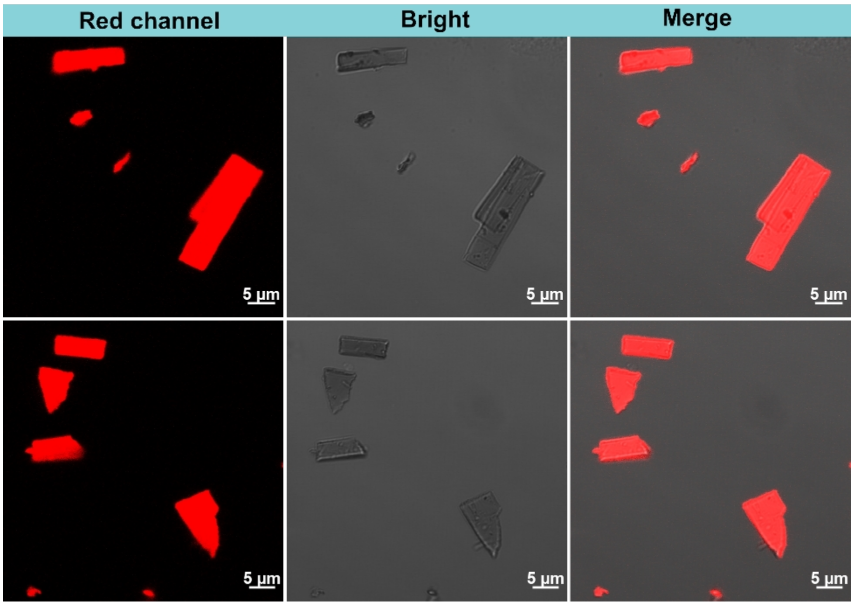

Supplementary Figure 22. **The morphology of TPE-PyT-CPS in DMEM.** Confocal microscopy imaging of TPE-PyT-CPS (20 μM) in dulbecco's modified eagle medium (DMEM) with different sizes. The experiments were performed three times independently, representative images are shown. Scale bar: 5 μm.

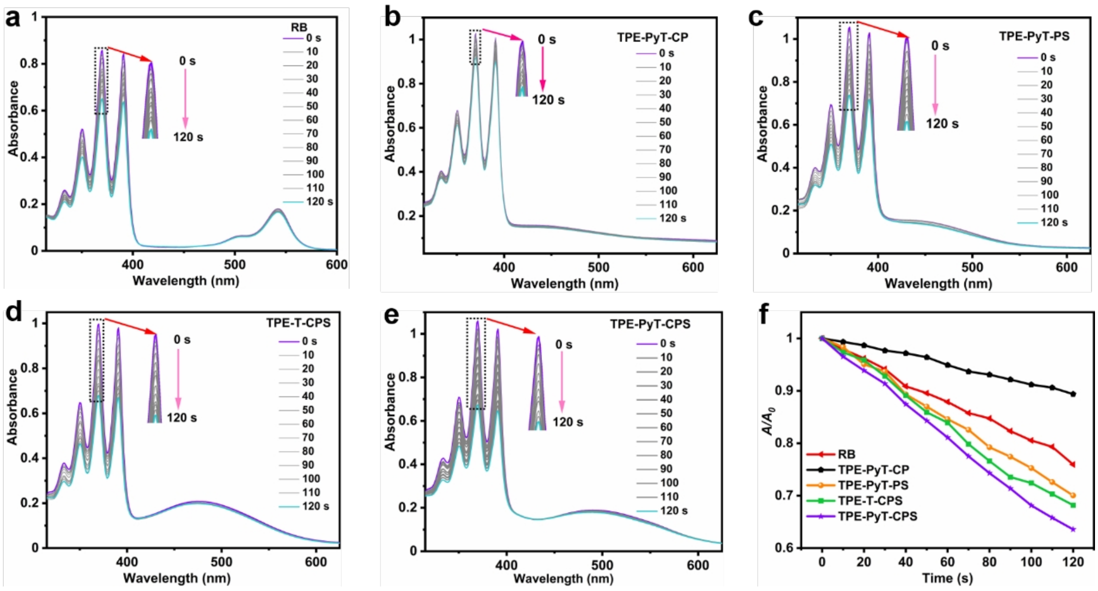

Supplementary Figure 23. **ROS generation capacity of the AIEgens in the presence of ABDA under light irradiation.** Changes of UV-vis spectra of ABDA (60 μM) in the presence of (a) RB (10 μM) and different AIEgens (10 μM) including TPE-PyT-CP (b), TPE-PyT-PS (c), TPE-T-CPS (d) and TPE-PyT-CPS (e) under different durations of light irradiation (10 mW cm^{-2}). (f) The decomposition rates of ABDA in different AIEgens under light irradiation (A_0 and A represent the absorption of ABDA before and after laser irradiation, respectively). The experiments in a-f were performed two times independently with similar results, representative images are shown. Source data are provided as a Source Data file.

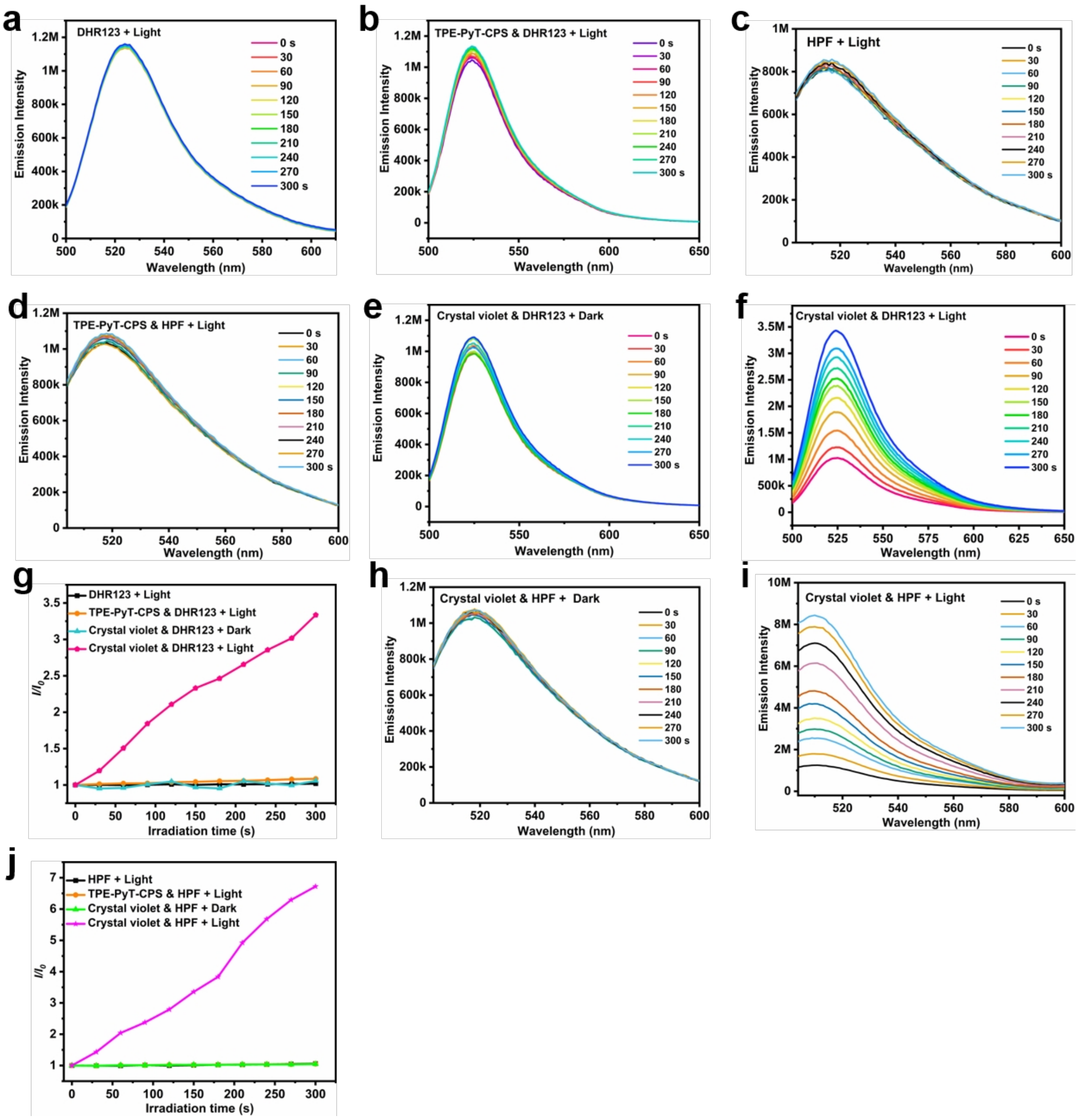

Supplementary Figure 24. HPF and DHR123 were used to verify the type I photosensitizer properties of TPE-PyT-CPS. ROS generation of TPE-PyT-CPS (1μM) and crystal violet (CV, 1μM) upon 532 nm laser irradiation in the presence of HPF (superoxide anion probe) or DHR123 (hydroxyl radical probe). DHR123 (a) and HPF (c) in the presence of light irradiation (532 nm laser,10 mW cm^{-2}). TPE-PyT-CPS in the presence of DHR123 (b) or with HPF (d) under light irradiation (532 nm laser,10 mW cm^{-2}). Crystal violet without (e) or with (f) light irradiation in the presence of DHR123 (532 nm laser,10 mW cm^{-2}). (g) The relative changes in PL intensity of DHR123 in the presence of TPE-PyT-CPS or Crystal violet. Crystal violet without (h) or with (i) light irradiation in the presence of HPF (532 nm laser,10 mW cm^{-2}). (j) The relative changes in PL intensity of HPF in the presence of TPE-PyT-CPS or Crystal violet. The experiments in a-j were performed two times independently with similar results, representative images are shown. Source data are provided as a Source Data file.

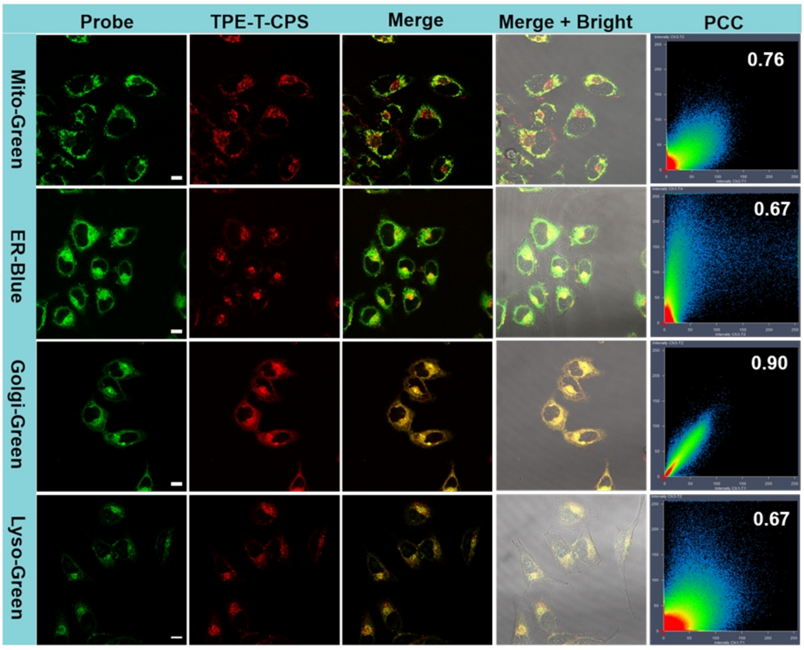

Supplementary Figure 25. Subcellular co-localization of TPE-T-CPS. CLSM of HeLa cells stained with different commercial probes (Mito-Green: 495-535 nm, λ_{ex}, 488 nm. ER-Blue:455-520 nm, λ_{ex}, 405 nm. Golgi-Green: 495-535 nm, λ_{ex}, 488 nm. Lyso-Green:495-535 nm, λ_{ex}, 488 nm) and TPE-T-CPS (10 μM, 600-750 nm, λ_{ex}, 488 nm). The experiments were performed three times independently, representative images are shown. Scale bar: 10 μm.

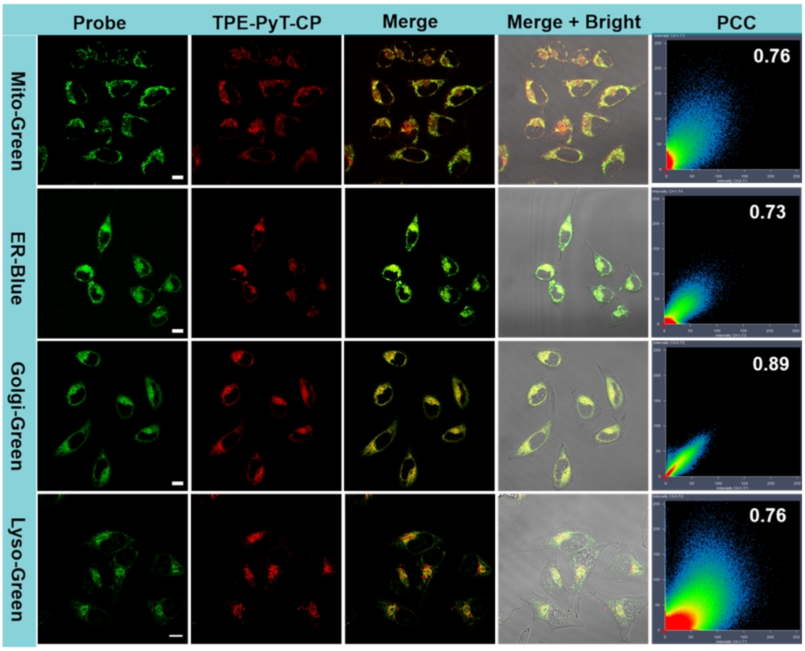

Supplementary Figure 26. Subcellular co-localization of TPE-PyT-CP. CLSM of HeLa cells stained with different commercial probes (Mito-Green: 495-535 nm, λ_{ex}, 488 nm. ER-Blue:455-520 nm, λ_{ex}, 405 nm. Golgi-Green: 495-535 nm, λ_{ex}, 488 nm. Lyso-Green:495-535 nm, λ_{ex}, 488 nm) and TPE-PyT-CP (10 μM, 600-750 nm, λ_{ex}, 488 nm). The experiments were performed three times independently, representative images are shown. Scale bar: 10 μm.

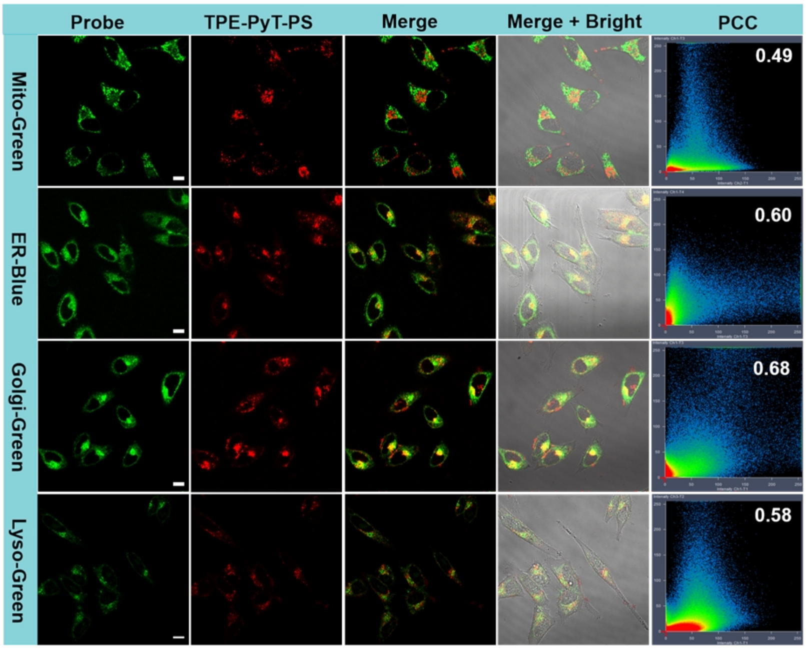

Supplementary Figure 27. Subcellular co-localization of TPE-PyT-PS. CLSM of HeLa cells stained with different commercial probes (Mito-Green: 495-535 nm, λ_{ex}, 488 nm. ER-Blue:455-520 nm, λ_{ex}, 405 nm. Golgi-Green: 495-535 nm, λ_{ex}, 488 nm. Lyso-Green:495-535 nm, λ_{ex}, 488 nm) and TPE-PyT-PS (10 µM, 600-750 nm, λ_{ex}, 488 nm). The experiments were performed three times independently, representative images are shown. Scale bar: 10 µm.

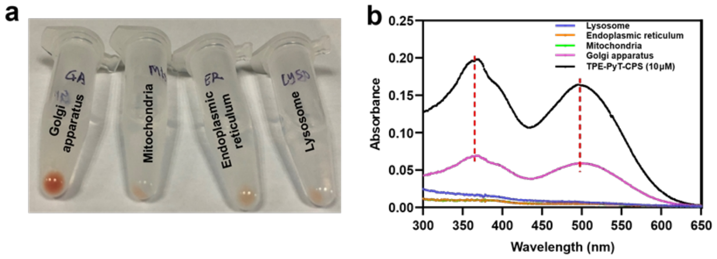

Supplementary Figure 28. Quantitative analysis of TPE-PyT-CPS in each subcellular organelle. (a) Picture of the TPE-PyT-CPS containing in each subcellular organelle after separation by different suborganelle kits. (b) UV absorption spectrum of TPE-PyT-CPS in each subcellular organelle in the mixed solvent (methanol/chloroform = 1:1, containing 5 µl acetic acid/ml). (c) Absorption of TPE-PyT-CPS in different subcellular organelles. The experiments in a-c were performed two times independently with similar results, representative images are shown. Source data are provided as a Source Data file.

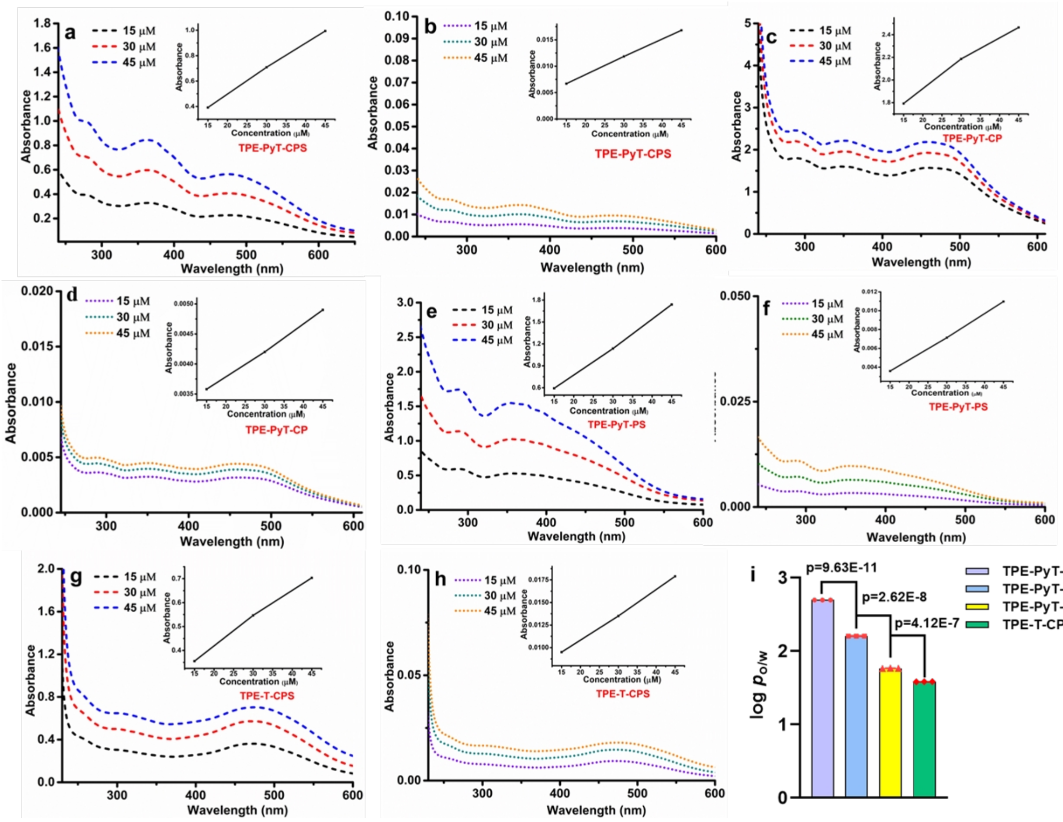

Supplementary Figure 29. Characterization the lipophilicity of the AIEgens. The UV spectrum of the AIEgens including TPE-PyT-CPS (a, b), TPE-PyT-CP (c, d), TPE-PyT-PS (e, f), TPE-T-CPS (g, h) in water and 1-octanol at different concentrations (15 µM, 20 µM, 45 µM): (a), (c), (e), (g) before shaking; (b), (d), (f) (h) after shaking. (i) Histogram of the log $P_{O/W}$ value of different AIEgens. Data in (i) are presented as mean ± SD derived from n = 3 independent samples (concentrations:15µM, 30µM, 45µM). Statistically significant differences between the experimental groups were analyzed by two-tailed Student's t-test. Source data are provided as a Source Data file.

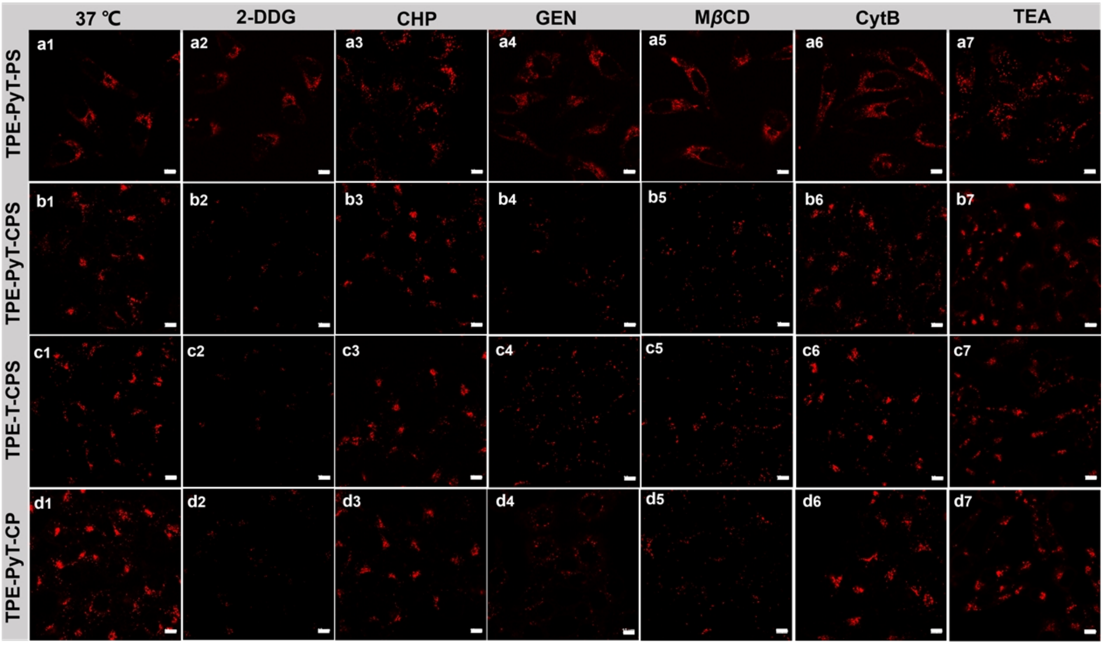

Supplementary Figure 30. Different inhibitors were used to analyze the uptake pathway of the AIEgens. CLSM images (Ex = 488 nm, Em = 600−750 nm) of HeLa cells incubated with the different AIEgens (10 μM) that contains TPE-PyT-PS (a1-a7), TPE-PyT-CPS (b1-b7), TPE-T-CPS (c1-c7), TPE-PyT-CP (d1-d7) in the presence of different cell uptake inhibitors including 2-DDG (ATP synthesis inhibitor), CHP (clathrin-mediated endocytosis inhibitor), GEN (caveolae-mediated endocytosis inhibitor), M*β*CD (lipid raft mediated endocytosis inhibitor), CytB (inhibitor of macropinocytosis) and TEA (organic cation transporters inhibitor). The experiments were performed three times independently, representative images are shown. Scale bar: 10 μm.

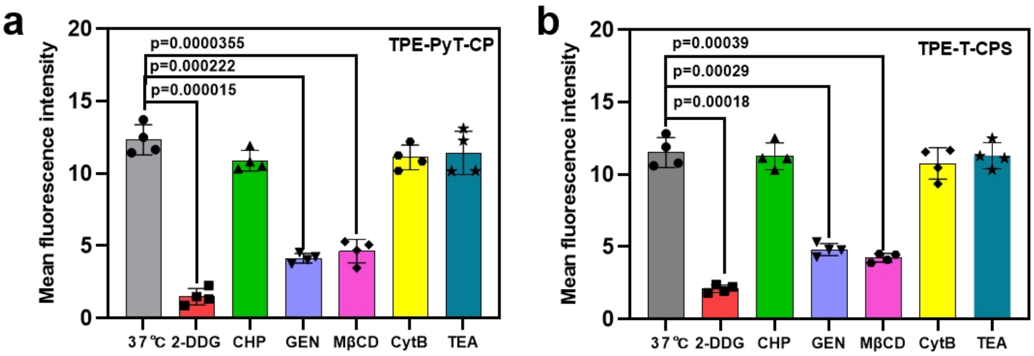

Supplementary Figure 31. The average fluorescence intensity of the AIEgens in cells was analyzed after treatment with different uptake inhibitor. Average fluorescence intensity of HeLa cells incubated with AIEgens including TPE-PyT-CP (a) and TPE-T-CPS in the presence of different endocytosis inhibitors including 2-DDG, CHP, GEN, M*β*CD, CytB and TEA. Data are presented as mean ± SD derived from n = 4 biologically independent experiments. Statistically significant differences between the experimental groups were analyzed by two-tailed Student's t-test. When $p < 0.05$, it was considered to have statistical significance. Source data are provided as a Source Data file.

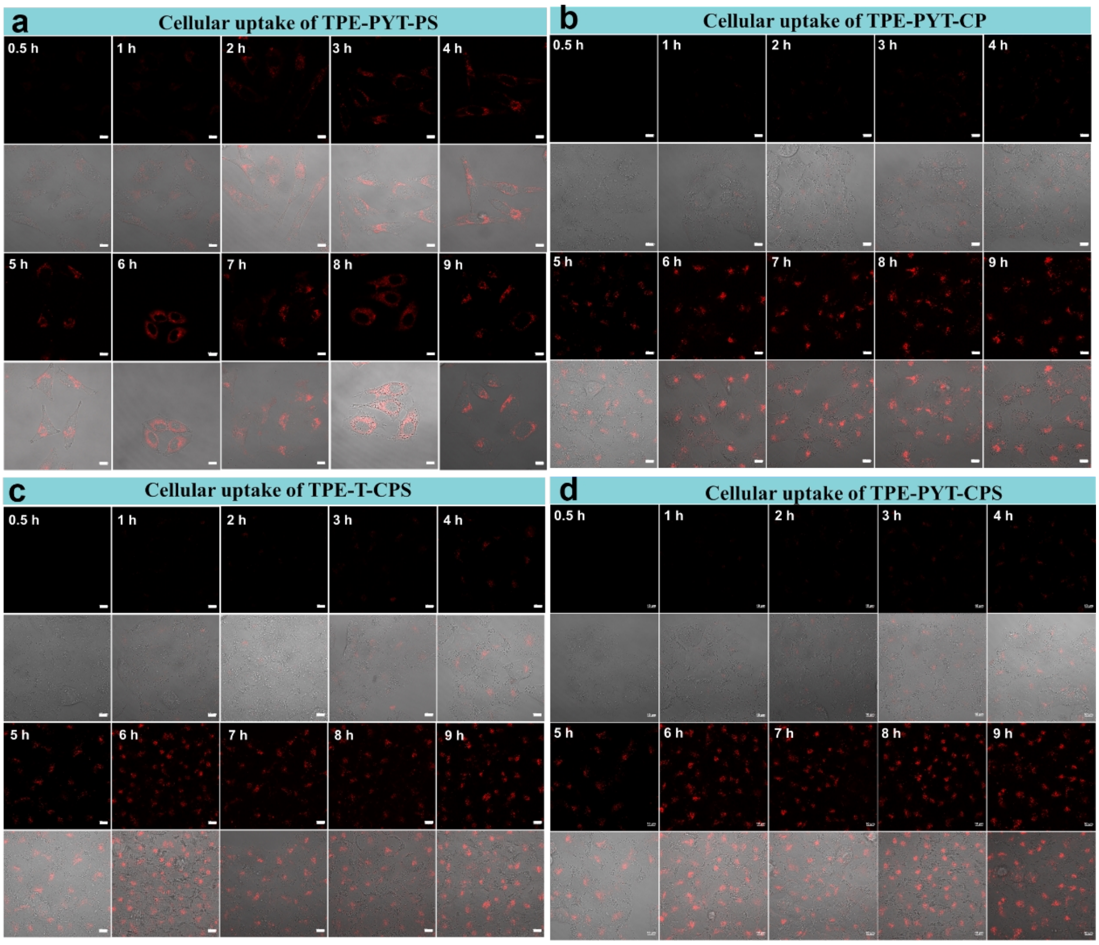

Supplementary Figure 32. CLSM images of the cell uptake with different AIEgens (10 μM) at 37 °C. CLSM images of different AIEgens including TPE-PyT-PS (a), TPE-PyT-CP (b), TPE-T-CPS (c) and TPE-PyT-CPS (d) in HeLa cells after co-incubation with HeLa cells for different times from 0.5 h to 9 h. The experiments were performed three times independently, representative images are shown. Scale bar: 10 μm.

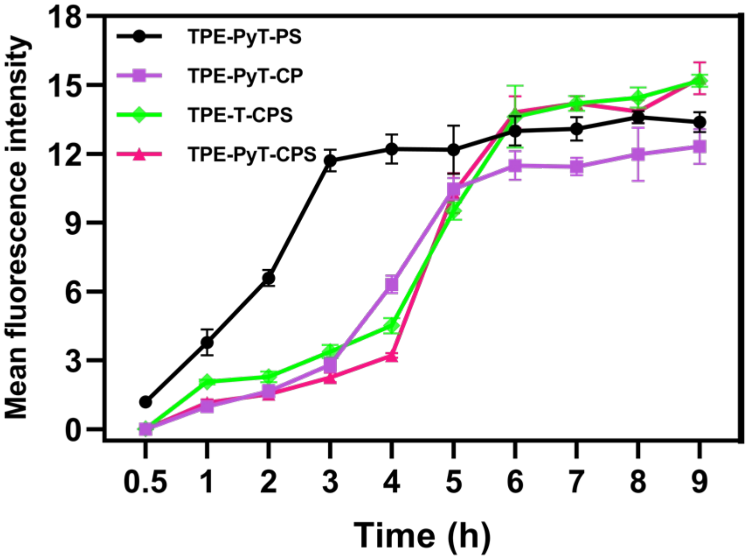

Supplementary Figure 33. The average fluorescence intensity of the AIEgens in cells was analyzed after co-incubation with HeLa cells for different times. Data are presented as mean ± SD derived from n = 3 biologically independent experiments. Source data are provided as a Source Data file.

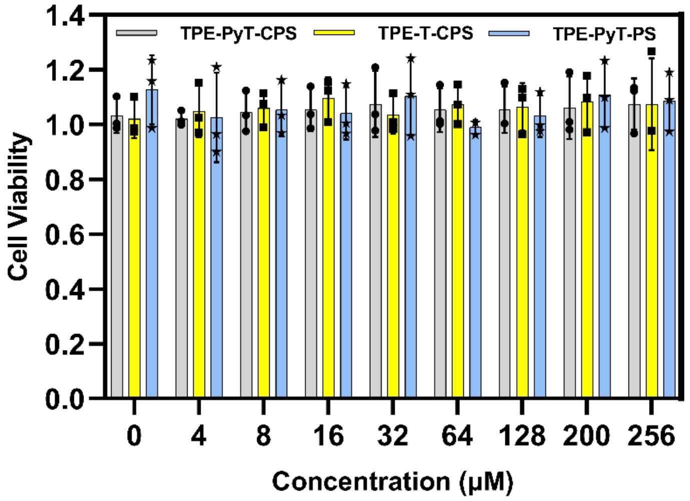

Supplementary Figure 34. Cell viabilities of HeLa cells after incubation with varied concentrations of AIEgens without 532 nm laser irradiation (dark). Data are presented as mean ± SD derived from n = 3 biologically independent experiments. Source data are provided as a Source Data file.

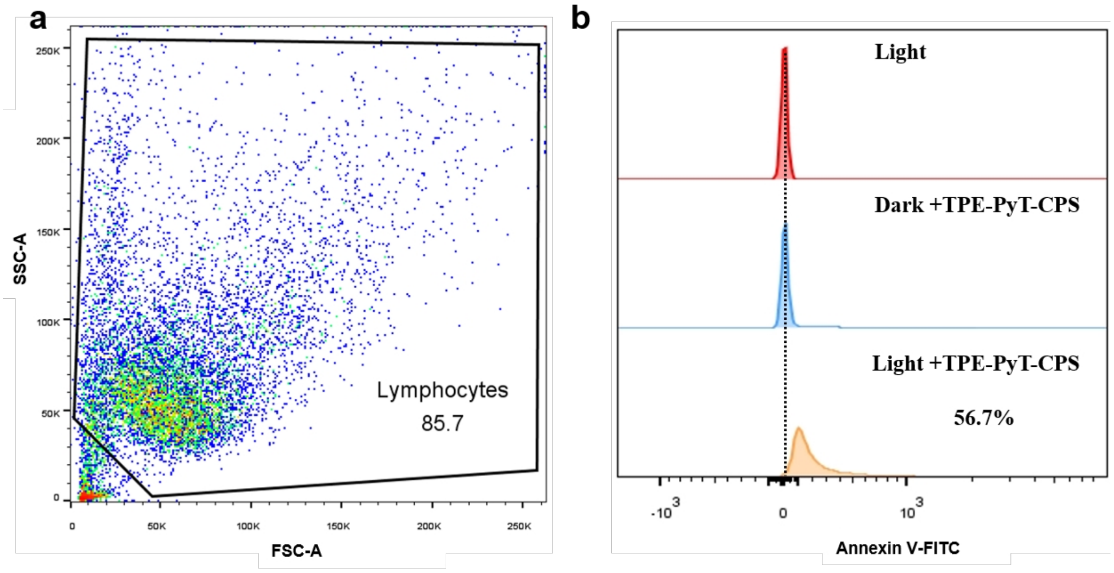

Supplementary Figure 35. (a) Gating strategies for flow cytometry analysis the apoptosis of HeLa cells in light group. (b) Apoptosis of HeLa cells after treatment with TPE-PyT-CPS (0.2 μM) under light irradiation (65 mW cm^{-2}, 2 min) and measured by using Annexin V in combination with flow cytometry.

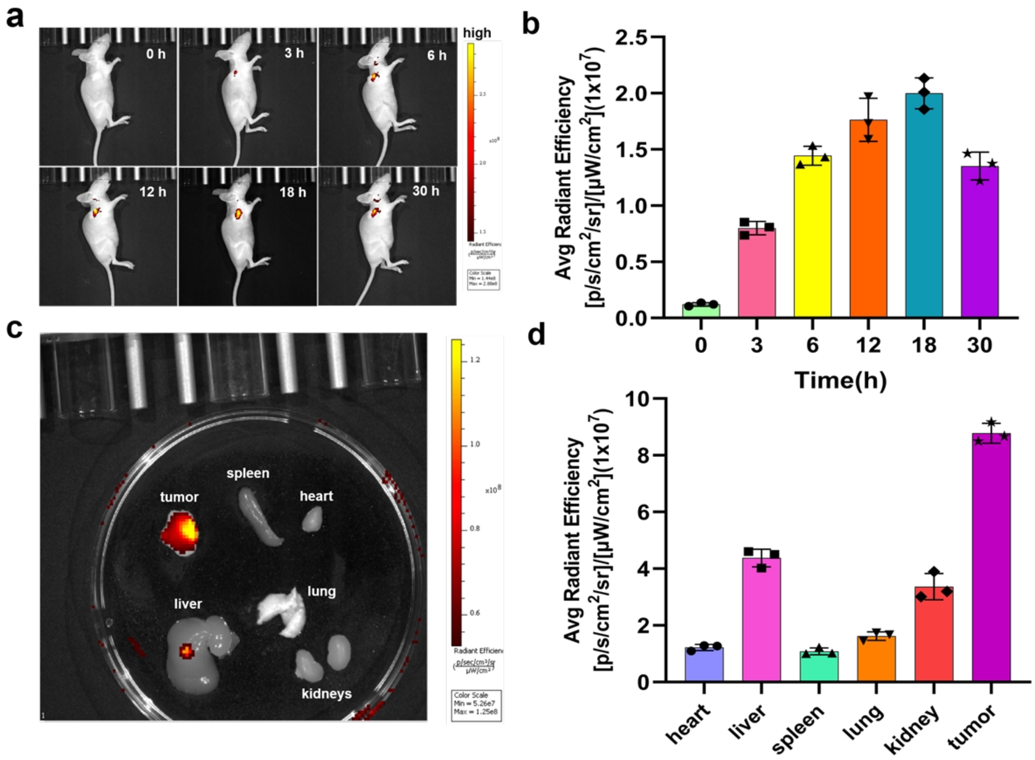

Supplementary Figure 36. The distribution of TPE-PyT-CPS in mice was studied by in vivo imaging. (a) Representative in vivo fluorescent images of TPE-PyT-CPS in HeLa-tumor-bearing mice after intratumoral injection of TPE-PyT-CPS (100 μM, 120 μL) at different time from 0 h to 30 h. (b) Quantification fluorescence intensity of TPE-PyT-CPS in mice tumor (n=3 individual animals). (c) Representative ex vivo fluorescence images and (d) average FL intensities of main organs resected from the mice after 18 h intratumoral injection of TPE-PyT-CPS (n=3 individual animals). The fluorescence images were acquired with λex/em = 530/690 nm. Data in (b) and (d) are presented as mean ± SD derived from n = 3 biologically independent animals. Source data are provided as a Source Data file.

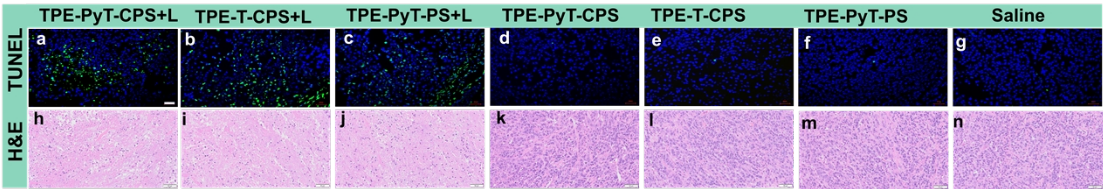

Supplementary Figure 37. The images of H&E and TUNEL staining of tumor tissue. Representative images of H&E staining (a-g) and apoptotic analysis of TUNEL staining (h-n) from the tumor tissues of mice in different treatments groups. The experiments of H&E and TUNEL staining were performed from biologically independent animals (n = 3), representative images are shown. Scale bar: 20 μm for TUNEL staining; 50 μm for H&E staining.

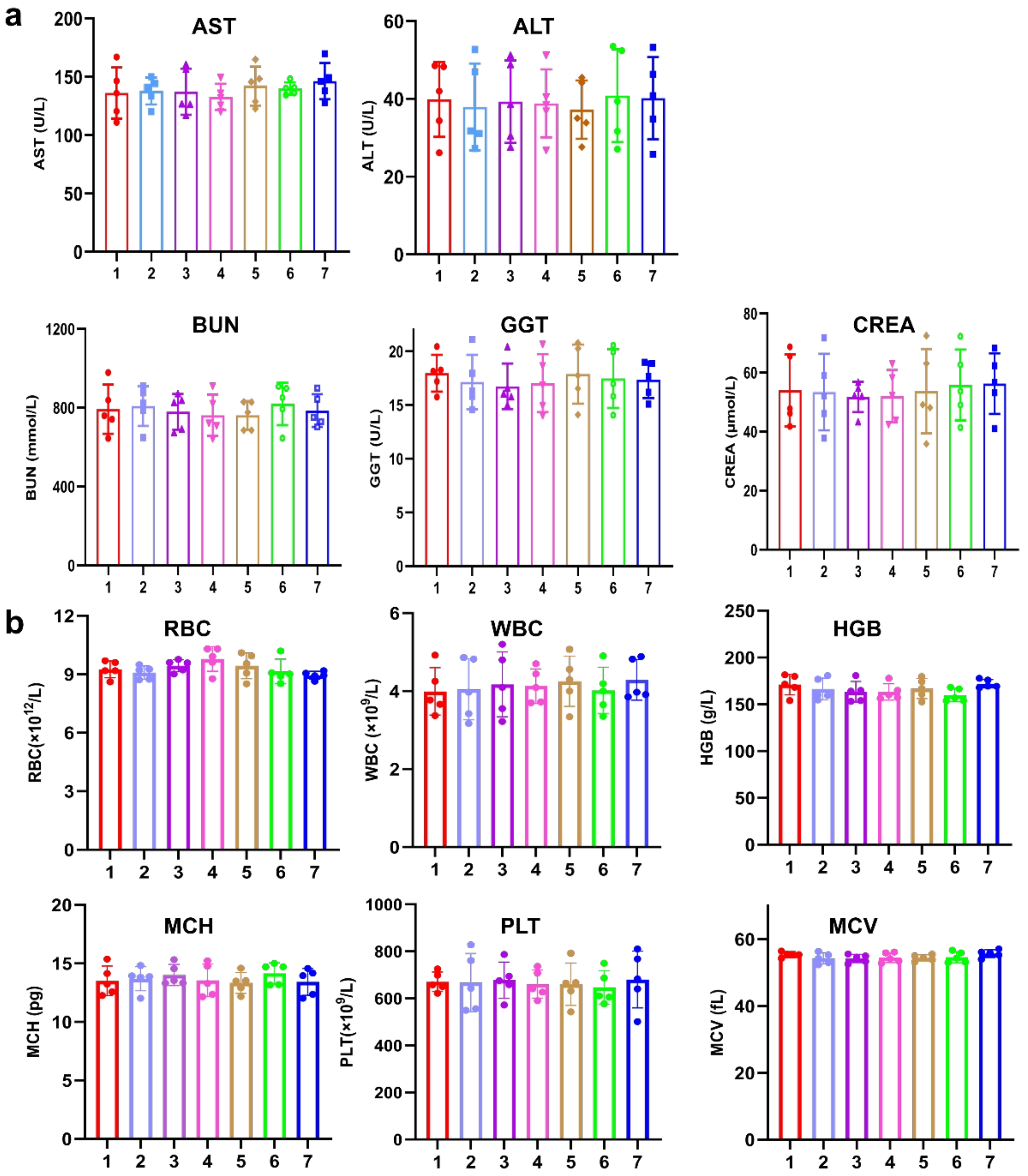

Supplementary Figure 38. Investigation of the biosafety of AIEgens in mice after PDT. (a) Biochemistry and (b) Blood count analysis of mice after photodynamic therapy in different treating group (1. saline + L, 2.TPE-PyT-PS, 3.TPE-T-CPS, 4.TPE-PyT-CPS, 5.TPE-PyT-PS + L, 6.TPE-T-CPS + L, 7.TPE-PyT-CPS + L). Abbreviations: AST, glutamic oxaloacetic transaminase; ALT, alanine aminotransferase; BUN, blood urea nitrogen; GGT, γ-glutamyl transpeptidase; CRE, creatinine; RBC, red blood cells; WBC, white blood cells; HGB, hemoglobin; MCH, mean corpuscular hemoglobin concentration; PLT, platelet count; MCV, mean corpuscular volume. Data are presented as mean ± SD derived from n = 5 biologically independent animals. Source data are provided as a Source Data file.

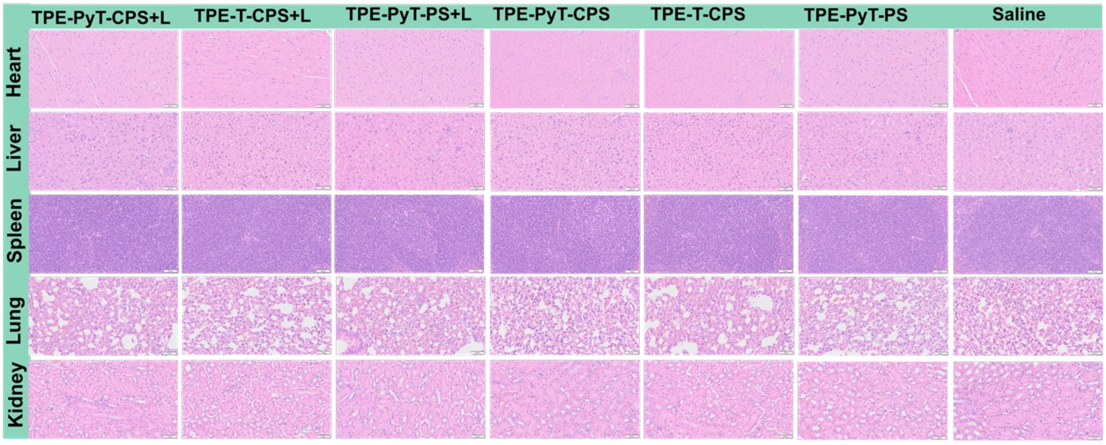

Supplementary Figure 39. Representative images of H&E-stained tumor and organ slices from mice including heart, liver, spleen, lung and kidneys from different treatment groups of Saline, TPE-PyT-PS, TPE-TC-PS, TPE-PyT-CPS, TPE-PyT-PS + L, TPE-T-CPS + L and TPE-PyT-CPS + L, respectively. The experiments were performed from biologically independent animals (n = 3), representative images are shown. Scar bar, 50 μm.

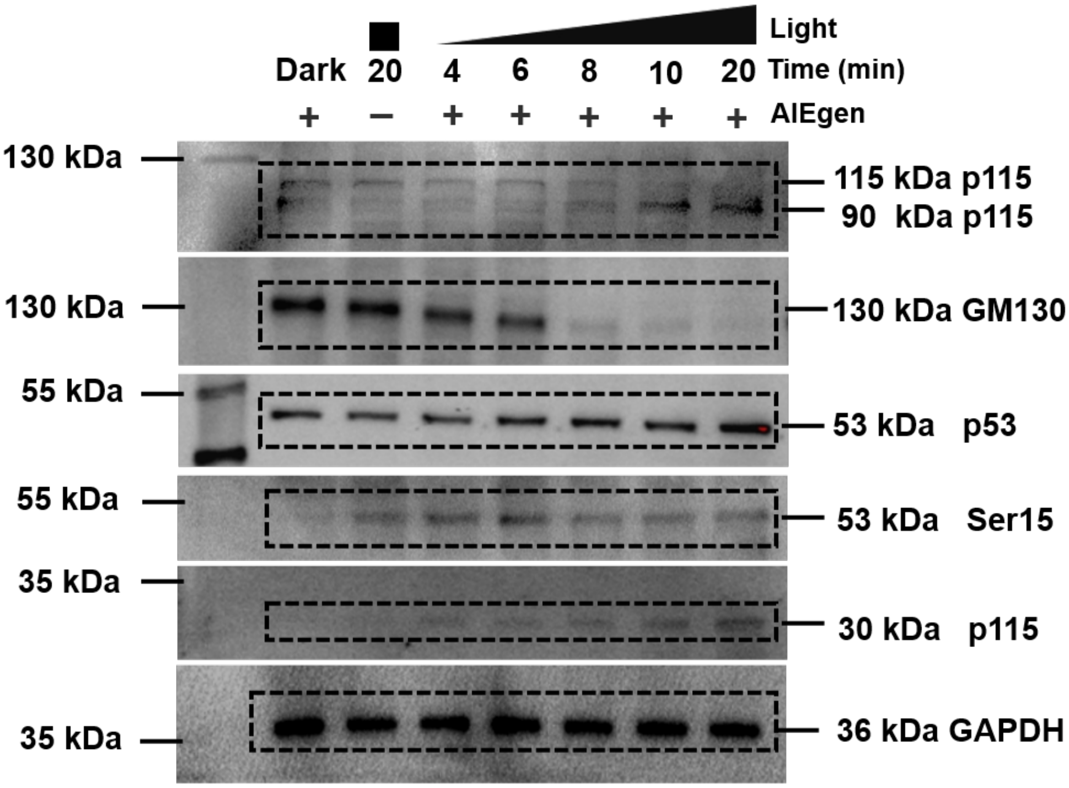

Supplementary Figure 40. Uncropped scans of western blot with molecular weight markers, it is related with Figure 7a.

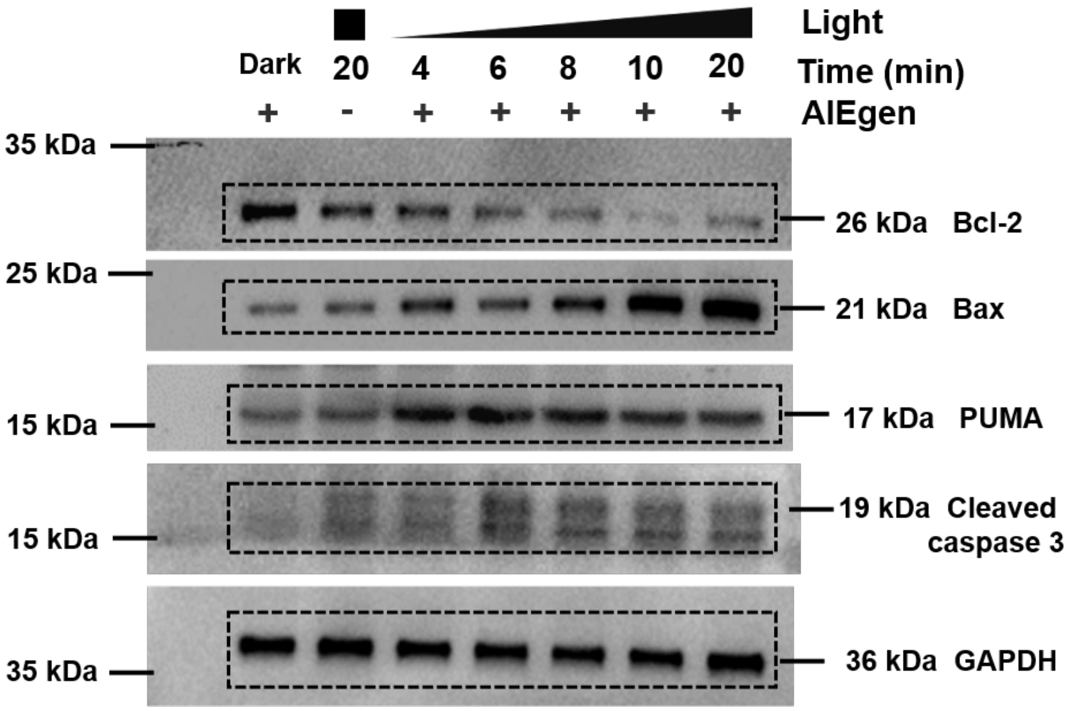

Supplementary Figure 41. Uncropped scans of western blot with molecular weight markers, it is related with Figure 7c.

Supplementary Table 1. Optical properties and lipophilicity of AIEgens including TPE-PyT-CP, TPE-PyT-PS, TPE-T-CPS, TPE-PyT-CPS.

AIEgens	$\lambda_{abs}{}^{a}$ (nm)	λ_{em} (nm)		$\Phi_f{}^{c}$ (%)			^{d}Log $P_{O/W}$
		Water^{b}	Solid state	Water	ACN	Solid state	
**TPE-PyT-CP**	428	625	703	12.1	1.28	2.79	2.6938 ± 0.0011
**TPE-PyT-PS**	438	653	704	13.6	0.78	4.95	2.2015 ± 0.0012
**TPE-T-CPS**	472	674	748	19.8	1.71	5.25	1.5808 ± 0.0012
**TPE-PyT-CPS**	487	680	756	17.1	2.74	5.63	1.7636 ± 0.0021

(a) Absorption maximum in water (containing 1% acetonitrile). (b) Emission maximum in water (containing 1% acetonitrile). (c) Fluorescence quantum yield of AIEgens in water, acetonitrile and solid state. (d) Data are presented as mean ± SD derived from n = 3 different concentrations (15µM, 30µM, 45µM) samples. Source data are provided as a Source Data file.

Supplementary Table 2. Selected parameters[a] for the calculated singlet state energy level and triplet state energy level of TPE-T-CPS and TPE-PyT-CPS.

Compound	States	Elecreonic transition	Energy, eV	f^b	Composition[c]	CI[d]
TPE-T-CPS	Singlet state	$S_0 \rightarrow S_1$	1.51	0.927	H→L	0.659
		$S_0 \rightarrow S_2$	2.34	0.032	H-1→L	0.695
		$S_0 \rightarrow S_3$	2.51	0.011	H-2→L	0.656
	Triplet state	$S_0 \rightarrow T_1$	1.11	0.000	H→L	0.658
		$S_0 \rightarrow T_2$	1.73	0.000	H-2→L	0.597
		$S_0 \rightarrow T_3$	2.16	0.000	H-3→L	0.494
TPE-PyT-CPS	Singlet state	$S_0 \rightarrow S_1$	1.32	0.807	H→L	0.705
		$S_0 \rightarrow S_2$	1.77	0.115	H-1→L	0.633
		$S_0 \rightarrow S_3$	2.10	0.002	H-2→L	0.706
	Triplet state	$S_0 \rightarrow T_1$	1.14	0.000	H→L	0.490
		$S_0 \rightarrow T_2$	1.41	0.000	H-2→L	0.505
		$S_0 \rightarrow T_3$	1.87	0.000	H-3→L	0.148

(a) Parameters were calculated by TD-DFT//B3LYP/6-31G(d), based on the optimized ground-state geometries using the method DFT//B3LYP/6-31G(d). (b) Oscillator strength. (c) H, HOMO (highest occupied molecular orbital) and L, LUMO (lowest unoccupied molecular orbital). (d) Coefficient of the wavefunction for each excitation.

Supplementary Table 3. Attenuation rate of AIEgens, RB and ICG after laser irradiation for 30 min (A_0: Photosensitizer before irradiation; A: Photosensitizer before irradiation).

Absorbance	TPE-PyT-CPS	TPE-PyT-PS	TPE-T-CPS	TPE-PyT-CP	Rose Bengal	Indocyanine Green
Maximum absorption	489 nm	429 nm	473 nm	427 nm	540 nm	770 nm
A_0	0.21277	0.13854	0.21559	0.16265	0.86336	1.59787
A	0.20785	0.13525	0.20851	0.15589	0.79108	1.38404
Attenuation rate: A_0-A / A	2.31 %	2.37 %	3.28 %	4.15 %	8.37 %	13.38 %
