# Supplementary material for: Golgi apparatus-targeted aggregation-induced emission luminogens for effective cancer photodynamic therapy
Source: Nat Commun. 2022 Apr 21;13:2179. doi: 10.1038/s41467-022-29872-7 (PMC9023483; doi:10.1038/s41467-022-29872-7)
Supplement: Supplementary file 2 — Reporting Summary [file 41467_2022_29872_MOESM2_ESM.pdf]

nature portfolio

Corresponding author(s): Yuncong Chen, Zijian Guo, Weijiang He

Last updated by author(s): March 16, 2022

Reporting Summary

Nature Portfolio wishes to improve the reproducibility of the work that we publish. This form provides structure for consistency and transparency in reporting. For further information on Nature Portfolio policies, see our Editorial Policies and the Editorial Policy Checklist.

Statistics

For all statistical analyses, confirm that the following items are present in the figure legend, table legend, main text, or Methods section.

n/a	Confirmed	
☐	☒	The exact sample size (n) for each experimental group/condition, given as a discrete number and unit of measurement
☐	☒	A statement on whether measurements were taken from distinct samples or whether the same sample was measured repeatedly
☐	☒	The statistical test(s) used AND whether they are one- or two-sided<br>*Only common tests should be described solely by name; describe more complex techniques in the Methods section.*
☐	☒	A description of all covariates tested
☐	☒	A description of any assumptions or corrections, such as tests of normality and adjustment for multiple comparisons
☐	☒	A full description of the statistical parameters including central tendency (e.g. means) or other basic estimates (e.g. regression coefficient) AND variation (e.g. standard deviation) or associated estimates of uncertainty (e.g. confidence intervals)
☐	☒	For null hypothesis testing, the test statistic (e.g. F, t, r) with confidence intervals, effect sizes, degrees of freedom and P value noted<br>*Give P values as exact values whenever suitable.*
☒	☐	For Bayesian analysis, information on the choice of priors and Markov chain Monte Carlo settings
☐	☒	For hierarchical and complex designs, identification of the appropriate level for tests and full reporting of outcomes
☐	☒	Estimates of effect sizes (e.g. Cohen's d, Pearson's r), indicating how they were calculated

Our web collection on statistics for biologists contains articles on many of the points above.

Software and code

Policy information about availability of computer code

Data collection	Perkin Elmer Lambda 35 spectrophotometer, FluoroMax-4(Horiba), Bruker Avance III 400 M NMR spectrometer, Agilent 6540 Q-TOF mass spectrometer, JEOL JEM-1011 transmission electron microscope (Japan), ZEISS Laser Scanning Microscope (LSM 710),BD LSRFortessa Cell Analyzer, PerkinElmer IVIS Lumina K Series III in vivo imaging system.
Data analysis	Mean fluorescence intensity (MFI) were obtained by Zen software on confocal microscope Zeiss LSM710. NMR spectra were analyzed using MestReNovel Lite software (Mestre Research S.L). Average area intensity of mice were recored by the Living Image software on PerkinElmer IVIS Lumina K Series III in vivo imaging system. Image J (Java 1.8.0_172) was used to analysis the western blot data. FlowJo_V10.6.2 was used to analyses the Flow Cytometry data. All data were analyzed and statistically calculated using Microsoft Excel 2016 software (Microsoft, Redmond, WA). All statistical data were presented as mean ± SD. The DFT calculations were performed with the Gaussian 09 W program package.

For manuscripts utilizing custom algorithms or software that are central to the research but not yet described in published literature, software must be made available to editors and reviewers. We strongly encourage code deposition in a community repository (e.g. GitHub). See the Nature Portfolio guidelines for submitting code & software for further information.

Data

Policy information about availability of data

All manuscripts must include a data availability statement. This statement should provide the following information, where applicable:

- Accession codes, unique identifiers, or web links for publicly available datasets
- A description of any restrictions on data availability
- For clinical datasets or third party data, please ensure that the statement adheres to our policy

The authors declare that the source data underlying Figures 2a-f, 4c-e, 5b-c, 7b, 7d, 10c-e and Supplementary Figures 18a-c, 19a-f, 20a-d, 21e-h, 23a-f, 24a-j, 29a-i, 31a-b, 33, 34, 36b, 36d, 38a-b are provided as a Source Data file. All other data supporting the findings of this study are available ether in the article and/or its Supplementary Information files.

Field-specific reporting

Please select the one below that is the best fit for your research. If you are not sure, read the appropriate sections before making your selection.

☒ Life sciences ☐ Behavioural & social sciences ☐ Ecological, evolutionary & environmental sciences

For a reference copy of the document with all sections, see nature.com/documents/nr-reporting-summary-flat.pdf

Life sciences study design

All studies must disclose on these points even when the disclosure is negative.

Sample size	Simple sizes of 2-5 biologically independent samples or animals per group were used for in vitro and in vitro studies, respectively, as indicated for specific experiments in Figure captions. We adhered to sample size requirements necessary for determining statistical significance with reference to the numbers used in recent relevant publications (Huang, Y., Guan, Z., Dai, X. et al. Nat .Commun 2021, 12, 2390 ; Zhang, W., Hu, X., Shen, Q. et al. Nat. Commun. 2019. 10, 1704)
Data exclusions	No data were excluded from the analyses.
Replication	All data presented were repeated for at least twice times with similar results as indicated in figure legends.
Randomization	The samples and animals used in this paper were randomly distributed into several groups for the further experiments.
Blinding	The experiment data were performed automatically with machine setups independently to the observer. Image acquisition and data analysis were automated and not subject to human bias. For quantitative analysis results (mean fluorescence intensity), we asked other researchers who were either involved or not involved in the study to examine blinded samples for biological effects. Occasionally blinding was deployed, as in tumor size measurement, with the measuring person having no knowledge on the identity of the mice being measured.

Reporting for specific materials, systems and methods

We require information from authors about some types of materials, experimental systems and methods used in many studies. Here, indicate whether each material, system or method listed is relevant to your study. If you are not sure if a list item applies to your research, read the appropriate section before selecting a response.

Materials & experimental systems

n/a	Involved in the study	
☐	☒	Antibodies
☐	☒	Eukaryotic cell lines
☒	☐	Palaeontology and archaeology
☐	☒	Animals and other organisms
☒	☐	Human research participants
☒	☐	Clinical data
☒	☐	Dual use research of concern

Methods

n/a	Involved in the study	
☒	☐	ChIP-seq
☐	☒	Flow cytometry
☒	☐	MRI-based neuroimaging

Antibodies

Antibodies used	Anti-p115-RhoGEF (Abcam, Catalog no: ab223759, 1:100 dilution,), Anti-GM130 (Abcam, Catalog no: ab52649, 1:1500 dilution), Anti-p53 (Abcam, Catalog no:ab26, 1:2000 dilution), Anti-p53 (phospho S15) (Abcam, Catalog no:ab1431, 1:1000 dilution), Anti-PUMA (Abcam, Catalog no:ab33906, 1:2000 dilution), Bcl-2 (Abcam, Catalog no:ab182858, 1:200 dilution), Bax (Abcam, Catalog no:ab32503, 1:5000 dilution), caspase-3 (Abcam, Catalog no:ab184787, 1:200 dilution) and Anti-GAPDH (Abcam, Catalog no:ab8245, 1:10000 dilution) were purchased from Abcam. Anti-rabbit HRP secondary antibody (Cell Signaling Technology, Catalog no:7074S, 1:2000

	dilution).
Validation	All antibody were verified by the supplier and each lot has been quality tested. Validation details of the antibodies are available on the manufacture's websites:<br>https://www.abcam.cn/puma-antibody-ep512y-ab33906.html<br>https://www.abcam.cn/p115-rhogef-antibody-ab223759.html.<br>https://www.abcam.cn/p53-phospho-s15-antibody-ab1431.html<br>https://www.cellsignal.cn/products/secondary-antibodies/anti-rabbit-igg-hrp-linked-antibody/7074?_=1642406733512&Ntt=7074&tahead=true<br>https://www.abcam.cn/gapdh-antibody-6c5-loading-control-ab8245.html<br>https://www.abcam.cn/caspase-3-antibody-epr18297-ab184787.html<br>https://www.abcam.cn/bax-antibody-e63-ab32503.html<br>https://www.abcam.cn/bcl-2-antibody-epr17509-ab182858.html<br>https://www.abcam.cn/p53-antibody-pab-240-ab26.html<br>https://www.abcam.cn/gm130-antibody-ep892y-cis-golgi-marker-ab52649.html.

Eukaryotic cell lines

Policy information about cell lines

Cell line source(s)	The human cervical cancer cell line HeLa were purchased from Stem Cell Bank, Chinese Academy of Science (Shanghai, China)
Authentication	Cell lines were authenticated by short tandem repeat (STR) testing.
Mycoplasma contamination	Cells lines were tested negative for mycoplasma contamination.
Commonly misidentified lines (See ICLAC register)	No commonly misidentified lines were used.

Animals and other organisms

Policy information about studies involving animals; ARRIVE guidelines recommended for reporting animal research

Laboratory animals	BALB/C-nu female male mice aged 6–8 weeks of specific pathogen free (SPF) grade were purchased from GemPharmatech Co., Ltd., license No.: SCXK (Jiangsu) 2018-0008. The mice were housed in animal-holding units in a pathogen-free environment with temperature at 22 ± 2 °C and 55 ± 5% humidity, under the 12 h/12 h dark/light cycle.
Wild animals	No wild animals were used in this work.
Field-collected samples	No field-collected samples were used in this work.
Ethics oversight	All the animal experiments in this study were approved by the Institutional Animal Care and Use Committees of GemPharmatech, approval No.: SYXK (Jiangsu) 2018-0027.

Note that full information on the approval of the study protocol must also be provided in the manuscript.

Flow Cytometry

Plots

Confirm that:

- [x] The axis labels state the marker and fluorochrome used (e.g. CD4-FITC).
- [x] The axis scales are clearly visible. Include numbers along axes only for bottom left plot of group (a 'group' is an analysis of identical markers).
- [ ] All plots are contour plots with outliers or pseudocolor plots.
- [x] A numerical value for number of cells or percentage (with statistics) is provided.

Methodology

Sample preparation	HeLa cells were seeded in a 6 cm plate (Corning) at a density of 2x105 cells mL−1 and incubated overnight to 60–70% confluence. Then the culture medium was replaced with medium containing TPE-PyT-CPS (0.2 µM). Photoirradiation was imposed with a 532 nm laser (65 mW cm−2, 2 min) after 6 h of incubation. Apoptosis assay was performed 24 h after irradiation. Cells were detached by trypsin and washed with PBS. The cell precipitation was suspended into the 1× binding buffer (100 µL) containing 5 µL Annexin V-FITC (BD Biosciences) and incubated at room temperature for 1 h in the dark. The analysis was performed by BD FACSCalibur flow cytometer within 1 h after adding 400 µL 1 × binding buffer.
Instrument	BD LSRFortessa Cell Analyzer
Software	FlowJo_V10

Cell population abundance	The flow cytometry was performed on each sample to a total cell number for at least 100000 events
Gating strategy	Briefly, the single cells were selected by FSC and SSC plot. The The fluorescence signal of annexin V was used as an index to detect apoptosis HeLa cells.

☒ Tick this box to confirm that a figure exemplifying the gating strategy is provided in the Supplementary Information.
